# Detection of focal source and arrhythmogenic substrate from body surface potentials to guide atrial fibrillation ablation

**DOI:** 10.1371/journal.pcbi.1009893

**Published:** 2022-03-21

**Authors:** Yingjing Feng, Caroline H. Roney, Jason D. Bayer, Steven A. Niederer, Mélèze Hocini, Edward J. Vigmond

**Affiliations:** 1 IHU Liryc, Electrophysiology and Heart Modeling Institute, fondation Bordeaux Université, Pessac-Bordeaux, France; 2 Univ. Bordeaux, IMB, UMR 5251, Talence, France; 3 School of Biomedical Engineering and Imaging Sciences, King’s College London, London, United Kingdom; 4 Bordeaux University Hospital (CHU), Electrophysiology and Ablation Unit, Pessac, France; Stanford University, UNITED STATES

## Abstract

Focal sources (FS) are believed to be important triggers and a perpetuation mechanism for paroxysmal atrial fibrillation (AF). Detecting FS and determining AF sustainability in atrial tissue can help guide ablation targeting. We hypothesized that sustained rotors during FS-driven episodes indicate an arrhythmogenic substrate for sustained AF, and that non-invasive electrical recordings, like electrocardiograms (ECGs) or body surface potential maps (BSPMs), could be used to detect FS and AF sustainability. Computer simulations were performed on five bi-atrial geometries. FS were induced by pacing at cycle lengths of 120–270 ms from 32 atrial sites and four pulmonary veins. Self-sustained reentrant activities were also initiated around the same 32 atrial sites with inexcitable cores of radii of 0, 0.5 and 1 cm. FS fired for two seconds and then AF inducibility was tested by whether activation was sustained for another second. ECGs and BSPMs were simulated. Equivalent atrial sources were extracted using second-order blind source separation, and their cycle length, periodicity and contribution, were used as features for random forest classifiers. Longer rotor duration during FS-driven episodes indicates higher AF inducibility (area under ROC curve = 0.83). Our method had accuracy of 90.6±1.0% and 90.6±0.6% in detecting FS presence, and 93.1±0.6% and 94.2±1.2% in identifying AF sustainability, and 80.0±6.6% and 61.0±5.2% in determining the atrium of the focal site, from BSPMs and ECGs of five atria. The detection of FS presence and AF sustainability were insensitive to vest placement (±9.6%). On pre-operative BSPMs of 52 paroxysmal AF patients, patients classified with initiator-type FS on a single atrium resulted in improved two-to-three-year AF-free likelihoods (p-value < 0.01, logrank tests). Detection of FS and arrhythmogenic substrate can be performed from ECGs and BSPMs, enabling non-invasive mapping towards mechanism-targeted AF treatment, and malignant ectopic beat detection with likely AF progression.

## Author summary

Atrial fibrillation (AF) is the most common cardiac arrhythmia, with focal sources of excitation being an important mechanistic cause, especially in paroxysmal AF patients. The standard treatment for AF, pulmonary vein isolation, confines focal source activity within the veins, but may not eliminate the risk of AF since it leaves others untreated. We hypothesized that non-invasive electrical recordings, such as standard 12-lead electrocardiograms and 252-lead body surface potentials, could provide useful information for therapy planning. We generated a synthetic dataset of 2973 AF episodes, driven or initiated by focal and non-focal sources in patient-specific atrial geometries, to train three mechanism-inspired classifiers which determined the presence and location of focal sources, as well as the sustainability of AF in atria without a focal source present. We showed the mechanistic basis of using the pre-operative signals to predict AF susceptibility after focal ablation. Our classifiers were accurate, efficient and robust to inter-patient variability on the synthetic dataset. By using their pre-operative recordings to estimate AF mechanisms for 52 paroxysmal AF patients, our classifiers predicted a patient subgroup with improved two-to-three-year AF-free rates post-ablation, demonstrating the potential to guide mechanism-directed treatment based on non-invasive signals.

## Introduction

Atrial fibrillation (AF) is the most common arrhythmia, and increases risks of stroke and heart failure. It is acknowledged that ectopic activities, such as focal sources (FS), can play an important role in AF, especially for paroxysmal AF patients [1]. FS refer to repetitive ectopic beats, which can either directly drive AF, or trigger the reentrant activities in AF. Ectopic beats can occur in young healthy subjects [2], and more frequently with aged people [3]. High counts of atrial ectopic beats measured by 24-hour or longer Holter electrocardiograms (ECGs) in asymptomatic subjects have been associated with higher risks of AF incidents [4–6]. However, as ectopic beats are also markers of other cardiac conditions associated with AF, such as stroke, the causality from frequent ectopic beats to AF incidents could not be established [7], indicating a need for other markers which enable mechanism-based prediction.

Electrical isolation or destruction of FS, both pulmonary vein (PV) and non-PV foci [1, 8], has been widely accepted as a standard AF treatment, especially for paroxysmal AF patients [9]. However, an arrhythmogenic substrate, which is not usually targeted by focal ablation procedures, can still render the atria susceptible to AF. A recent review [10] also proposed an AF electrophenotype spectrum that shifts from FS-dominant to more substrate-susceptible, as AF progresses. Because of this, the author also recommended pre-operative screening of the AF mechanism to direct personalized treatment, which has not been fully achieved non-invasively. Technologies that enable non-invasive identification of ablation targets, defined by patient AF mechanism, are therefore highly valuable. Moreover, the identification of malignant ectopic beats with likely AF progression potentially informs early intervention, which is associated with higher treatment success rates [11], as chronic AF results in tissue remodelling and fibrosis [12] which stabilize AF.

Both ECGs and body surface potential maps (BSPMs) acquire electric potential signals from multiple leads over the torso, and are common non-invasive mapping tools to detect cardiac abnormality. BSPMs have been analyzed to assess organization of AF by looking at frequency components [13]. They have also been used prior to, and during AF procedures, to compute epicardial phase maps to identify AF drivers via ECG imaging (ECGi) [14], which reduced the operation time [15]. However, although these non-invasive mapping technologies show promising results, the accuracy of ECGi has been primarily investigated during sinus rhythms or AF episodes perpetuated by a single driver [16], but not for more complex AF cases, due to limitation of the ECG imaging [17] and difficulty in simultaneously mapping atrial and surface potentials. For AF episodes, anything beyond dominant frequency (DF) [18] has not been validated.

To extract FS from body surface potentials, we exploit their characteristics of high periodicity with a short cycle length (CL) [19]. However, high periodicity is not a unique characteristic in itself as, in theory, it is also exhibited by stationary rotors or reentries. It is therefore essential to perform multi-source analysis for all periodic sources. Second-order blind source separation (SO-BSS) is a class of well-established methods to extract periodic sources from signals through maximizing the periodicity of sources. SO-BSS does not require a prior knowledge of the number of sources, which is usually a drawback for using blind source separation techniques in practice, such as FastICA [20], and the extracted sources can be ranked according to the periodicity of the source. Previously, SO-BSS methods have been used to extract atrial components from AF patient ECGs [21], separate fetal and maternal contributions from mother ECGs [22], as well as extract FS from AF patient intracardiac electrograms [23]. From multiple extracted sources, to classify the presence and the located chamber of FS, as well as the presence of arrhythmogenic substrate for sustained AF, we then adopted a nonlinear statistical model for high-dimensional features, a random forest classifier [24]. A random forest classifier is an ensemble of decision trees, where each node maximizes the information gain by splitting the parameter space of a feature. It then assembles these decision trees using boot-strap sampling, to reduce the risk of overfitting.

Hypothesizing that sustained rotors during FS-driven episodes suggest an arrhythmogenic substrate which can be detected non-invasively with SO-BSS, the objective of this study was to non-invasively detect the presence of FS and an AF-susceptible substrate, as well as the location of the focal site if it exists, in order to suggest appropriate ablation targets. We did so using computer simulations of realistic AF episodes driven by FS and/or reentrant sources, and by modelling focal ablation by removing FS, to form digital twins in training the classifiers [25]. AF inducibility was ascertained by looking at AF sustainability after the FS was removed. From potentials computed on the torso, we sought to determine the prediction targets by extracting features using SO-BSS, and feeding these into a random forest classifier. This may help to select appropriate ablation targets for AF patients, and detect the presence of malignant ectopic beats in the general population. All abbreviations used in the manuscript are listed in Table 1.

**Table 1:** Table of abbreviations.

Abbreviation	Full name
AF	Atrial Fibrillation
ACF	Auto-correlation function
ACh	Acetylcholine
APD	Action Potential Duration
AUROC	Area Under the Receiver Operating Characteristic Curve
BSPM	Body Surface Potential Map
CL	Cycle Length
DF	Dominant Frequency
ECG	Electrocardiogram
ERP	Effective Refractory Period
FFT	Fast Fourier Transform
FS	Focal Source(s)
LA	Left Atrium
MaxAC	Maximal Auto-Correlation
NDI	Non-Dipolar component Index
SO-BSS	Second-Order Blind Source Separation
PCA	Principal Component Analysis
PS	Phase Singularity
PV	Pulmonary Vein
RA	Right Atrium
s.d.	standard deviation
n.u.	normalized unit
a.u.	arbitrary unit

## Results

**Fig 1:**
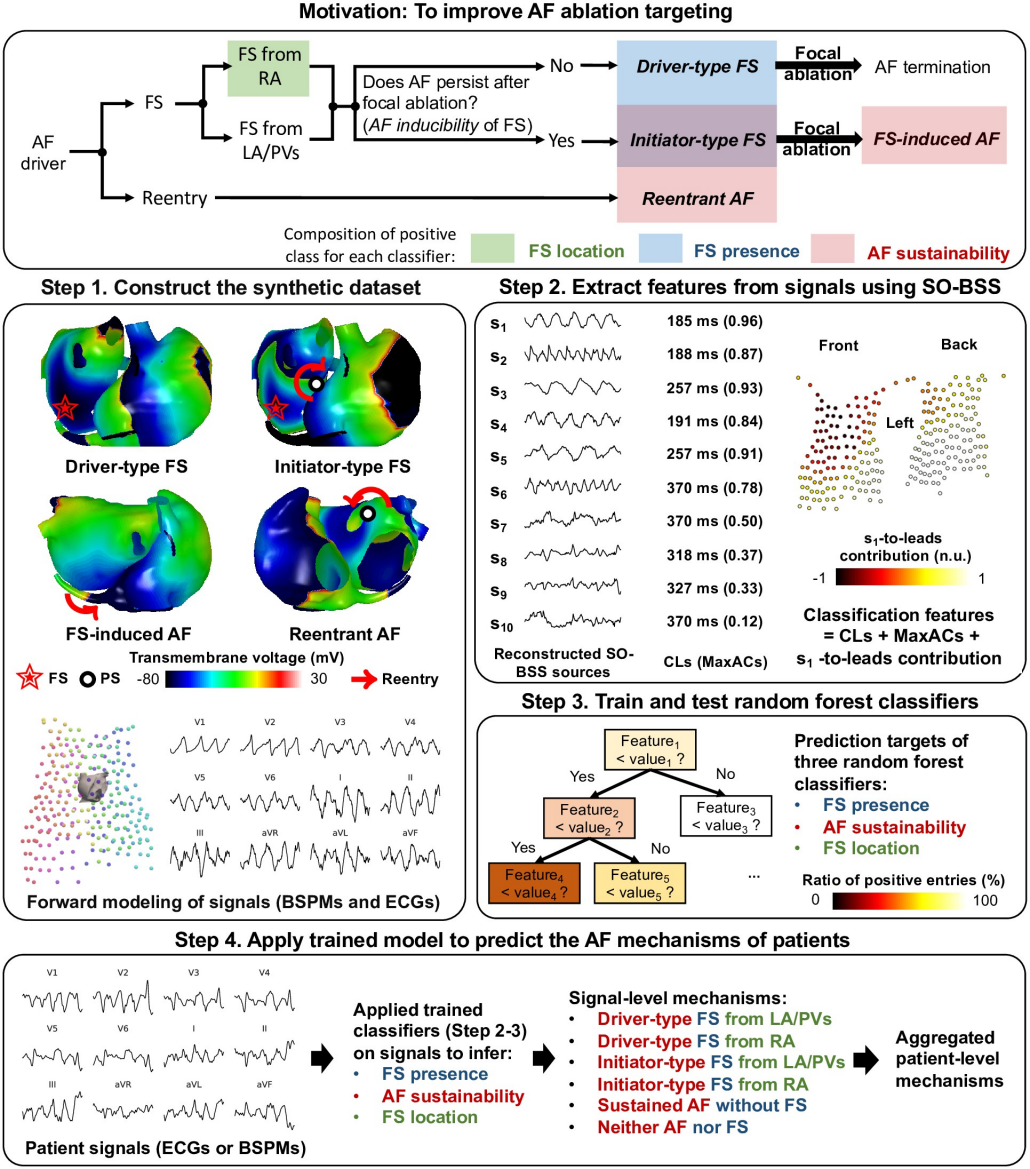
An overview of our methods. The motivation box illustrates the prediction of “FS presence” (shaded in blue), “FS location” (shaded in green), and “AF sustainability” (shaded in red) in selecting the ablation targets for AF, where shaded rectangles indicate the composition of the positive class for each classifier. Note that the initiator-type FS is in both classes of “FS presence” and “AF sustainability”. The bold italic fonts mark the four simulation categories in our synthetic dataset. Steps 1 to 3 describe the training process of our classifiers on synthetic data, and Step 4 illustrates the application of the trained classifiers on patient signals to non-invasively detect their AF mechanisms. In Step 1, white spheres show phase singularity (PS) points, the red arrows show the wavefront movement of rotors, and the red stars mark the focal sites.

We identified the classification of *FS presence*, *FS location* and *AF sustainability* as targets of AF ablation, as shown in the motivation box of Fig 1. The presence of FS requires its hinderance or removal for treatment, such as a focal ablation procedure. The localization of foci could speed up their mapping. An arrhythmogenic substrate suggests that the atria tissue can support sustained AF, implying a need for area ablation, such as modifying the substrate [26] by disrupting the pathway of reentrant drivers [14], and/or limiting the available area for rotor movement [27]. In the following sections, we denoted an FS as an *initiator-type FS* if AF was sustained after the focal ablation, and as a *driver-type FS* otherwise. AF after focal ablation for initiator-type FS was denoted as an *FS-induced AF*. A reentry driven AF episode was denoted as a *reentrant AF*. AF sustainability after removing the FS indicated *AF inducibility* from the FS on the atria.

### Analysis of simulated AF episodes

#### AF inducibility from FS

**Fig 2:**
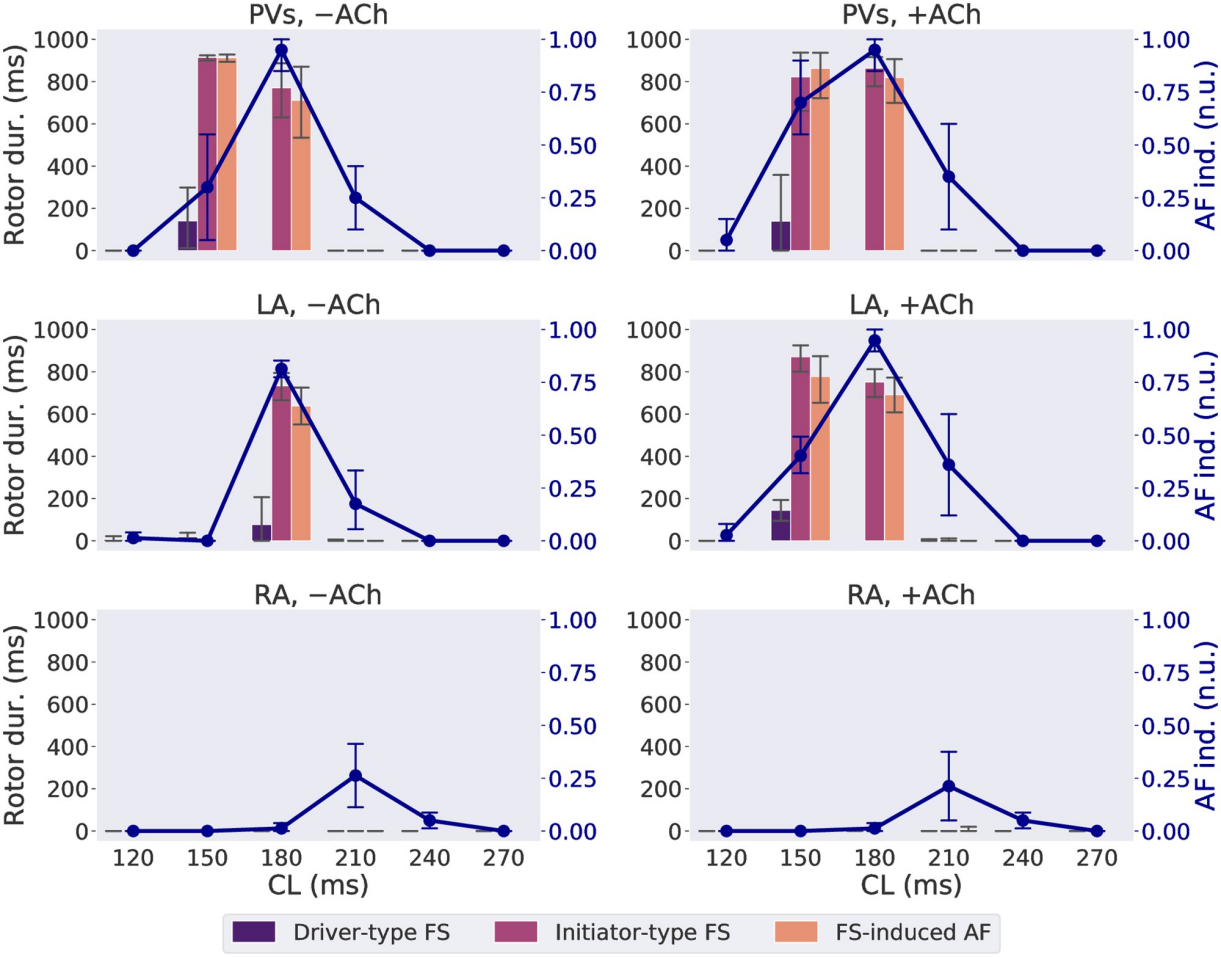
Rotor duration (“Rotor dur.” given by the bars) and AF inducibility (“AF ind.”, given by the line plot) as functions of CL of FS on LA, RA and PVs, with (“+”) or without (“−”) ACh. Vertical bars of the line plot show the 95% confidence interval of the mean of all patient meshes. CL groups accounting for less than 5% of FS in each panel were excluded from analysis and the bar charts. n.u.: normalized unit.

We systematically changed the CL and the location of FS, and the concentration of Acetylcholine (ACh) ([ACh]), across five patient atria, and calculated the AF inducibility and the rotor duration for each case of CL, focal location and [ACh] on each patient. The means and 95% confidence intervals across five patients are shown in Fig 2, and the increment of AF inducibility from FS by adding ACh regulation is shown in S1 Table.

AF inducibility was the highest from the PV FS, followed by the left atrium (LA) FS. The highest AF inducibility was observed from FS with a CL of 180 ms from the PVs and from the LA, with means 0.95 and 0.88, respectively, and with a CL of 210 ms from the right atrium (RA), with mean 0.24. The CL range with non-zero AF inducibility was also larger for FS from the PVs and the LA than in the RA. ACh regulation increased AF inducibility from FS with CLs of 150–210 ms from the LA, and from FS with a CL of 150 ms from the PVs, but not for FS from the RA.

For FS with the presence of sustained rotors, AF was more likely to be sustained than without. During FS-driven episodes, longer rotor duration was associated with higher AF inducibility, with an Area Under the Receiver Operating Characteristic Curve (AUROC) of 0.83. Rotor duration ≥ 100 ms occurred more frequently with initiator-type FS than driver-type FS (66.7% vs. 2.6%, p-value < 0.0001, one-sided proportional z-test). For FS with CLs of 150–180 ms from the LA and PVs, all cases where initiator-type FS had a longer rotor duration than driver-type FS were also associated with a high AF inducibility. The AF episodes induced by these initiator-type FS also had a similarly long rotor duration. The remaining 33.3% of initiator-type FS were driven by short-lived rotors or macro-reentries, mostly concentrated at a CL of 210 ms, regardless of the focal site. Over five patient meshes, FS at a CL of 210 ms showed a relatively high AF inducibility (mean 25.7%) and inter-patient variability (standard deviation (s.d.) ±18.5%), as shown in the second column of S2 Table.

**Fig 3:**
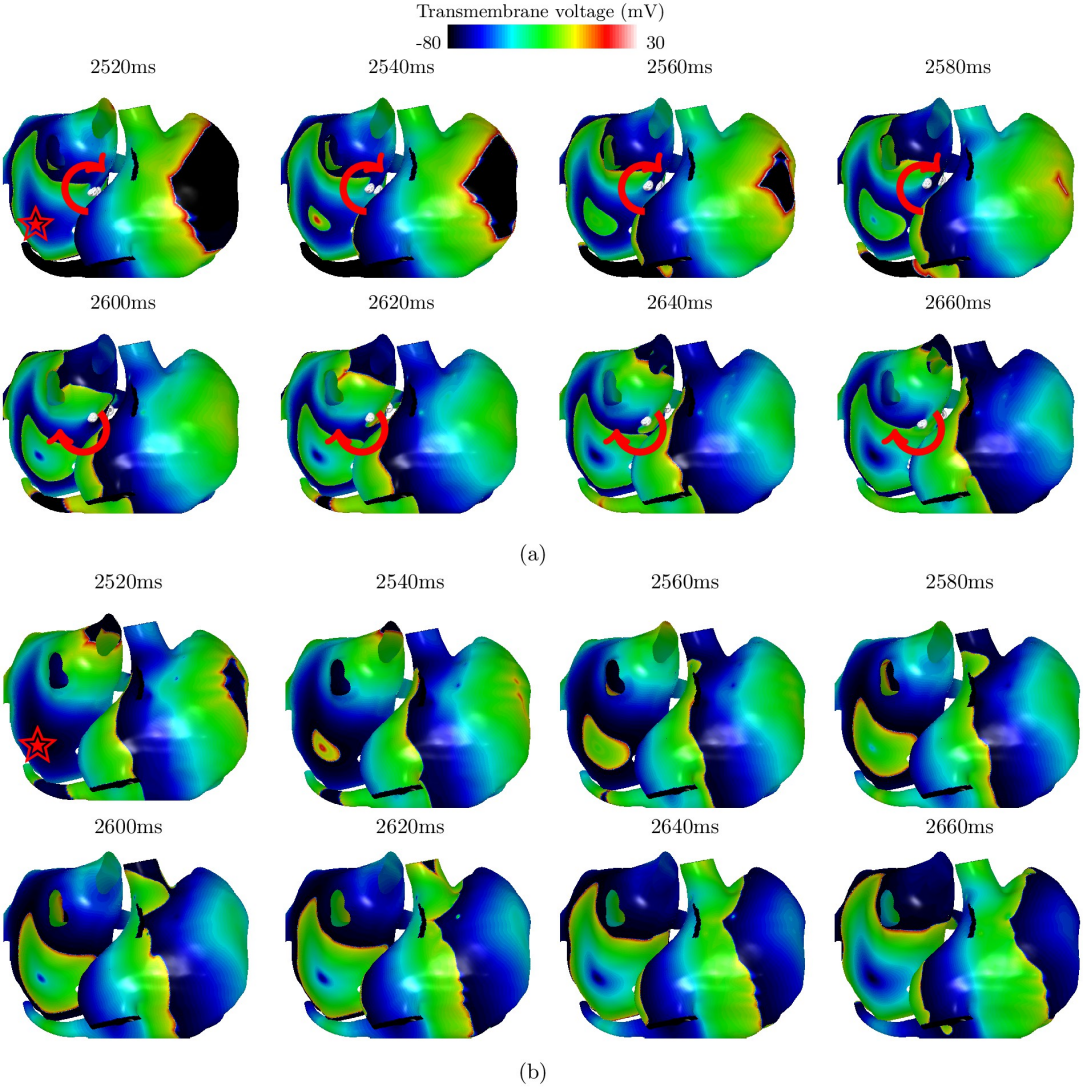
Simulations on the atrial mesh of Patient 2 of (a) an initiator-type FS with a CL of 180 ms at an LA focal site (*α*_*LA*_ = 0.2, *β*_*LA*_ = 0.2, red star), and (b) a driver-type FS with a CL of 210 ms at the same site (red star), with full videos (S1 and S2 Videos). The white spheres in (a) show phase singularity points, and the red arrows show the wavefront movement of rotors. The time since the first firing of the FS was indicated above each frame. A sustained rotor of total duration 547 ms was presented in (a), but no rotor was detected in (b).

For visualization, selected frames of an initiator-type FS and a driver-type FS are shown in Fig 3, with full videos (S1 and S2 Videos). Sustained rotors lasted for 547 ms in total during the initiator-type FS episode, but there was no sustained rotor in the driver-type FS episode. The effects of focal ablation on these FS are shown in S1 Fig, where the activation continued on the initiator-type FS, but not on the driver-type FS as a form of macro-reentry between the LA and the RA. Two reentrant AF episodes, including rotors and reentries around an unexcitable core of radius 1 cm, are shown in S2 Fig. The results of adding ACh regulation are shown in S3 Fig where small wavelets were located in areas with ACh islands densely distributed.

#### Factors that impact the AF-susceptible CL range

**Fig 4:**
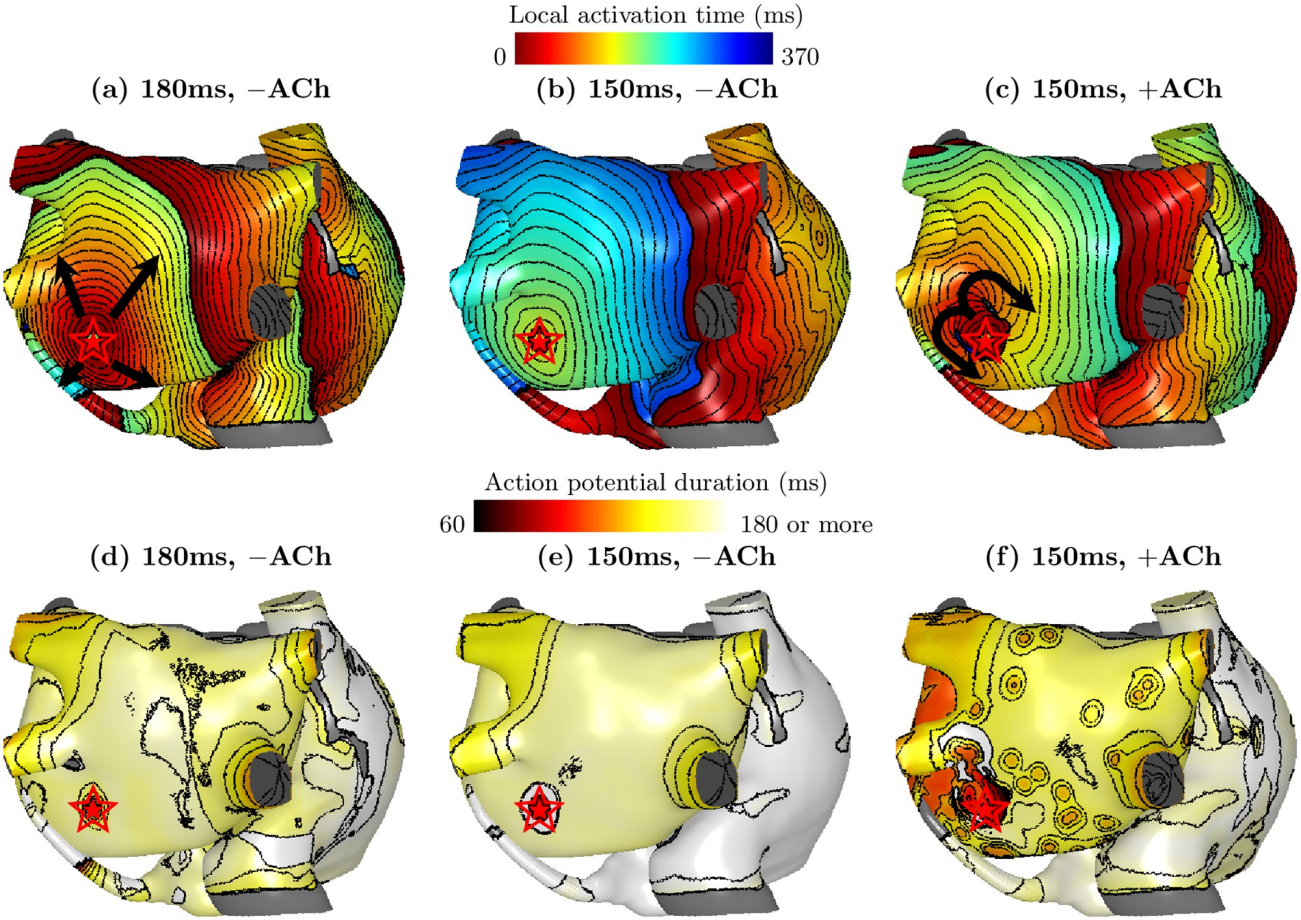
Local activation time maps (a–c) and action potential duration maps (d–f) following a discharge of an FS (red stars) from the same site on the LA posterior wall (*α*_*LA*_ = 0.4, *β*_*LA*_ = 0.2), on the atrial mesh of Patient 1, under focal cycle lengths of 150 ms and 180 ms, with (“+”) and without (“−”) ACh. The directions of wavefront propagation from the FS were shown by the black arrows. A gray color indicates tissue that was not excitable. Isochrone lines are drawn at an interval of 10ms. The focal activation was propagated at (a) and (c) but was blocked at (b). (f) shows the highest spatial heterogeneity in the action potential duration.

As can be seen from Fig 2, the AF-susceptible CL range varied between focal sites and [ACh]. To study the causes, the effective refractory period (ERP) was estimated by searching from long to short CLs for the boundary CL where the 1:1 atrial response with the FS disappears. As an example, as a complete conduction block was formed around the focus with CL = 150 ms (Fig 4b) but not with CL = 180 ms (Fig 4a), the ERP was estimated between 150 and 180ms. For the majority of cases without ACh, the ERP was estimated as 150–180 ms for the LA FS, and 180–210 ms for the RA FS, longer than the LA FS. Observing that the starting focal CL with high AF inducibility was also shorter for the LA FS than the RA FS from Fig 2, a correlation was seen between a shorter ERP and a shorter minimal focal CL required to induce AF.

In our simulations, ACh regulation shortened the refractory period, opening conduction pathways in some directions around an otherwise blocked focal discharge, which also amounts to a decrease in the ERP around the focal site. Furthermore, ACh regulation increased the spatial action potential duration (APD) heterogeneity of the atrial tissue, which provided a substrate for fibrillatory waves with variable CLs. Fig 4b shows a discharge of FS with CL = 150 ms on the LA without ACh, where the FS was completely blocked. Adding ACh in Fig 4c, unidirectional conduction blocks were seen around the focus, and a reentry was initiated. At the same time, a higher variability in the APD on the LA tissue was also observed with ACh in Fig 4f, compared to without ACh in Fig 4d and 4e.

### Non-invasive detection of FS and AF sustainability

#### Atrial source extraction with SO-BSS

To illustrate the advantages of SO-BSS, we showed examples of sources decomposed from BSPMs of Patient 2 with SO-BSS, in initiator-type FS (S4 Fig), driver-type FS (S5 Fig), and reentrant AF (S6 Fig), with their ECGs shown in S7 Fig. The ECG morphology is indistinguishable to human eyes between initiator-type FS (S7(a) Fig) and reentrant AF (S7(c) Fig). A Fast Fourier Transform (FFT) analysis also had a limited resolution of frequency (1 Hz) on a 1000 ms 1 kHz signal, where initiator-type FS and reentrant AF all showed the same DF of 4 Hz on the V1 lead. Their V1 signals also showed similar maximal values of ACF (0.68 and 0.67).

However, through extracting multi-source CLs, different degrees of CL alignment between SO-BSS sources were shown across different mechanisms. As the driver-type FS contained only one type of periodic source, CLs of extracted sources were the most uniform in the driver-type FS in nine out of ten sources. The sources of an initiator-type FS were less synchronized, with six (**s**_1_, **s**_2_, **s**_4_, **s**_6_, **s**_7_ and **s**_10_) that were synchronized (absolute difference ≤ 10 ms). The reentrant AF, however, had only three synchronized sources (**s**_1_, **s**_3_ and **s**_6_) with CLs similar to the dominant CL of 191 ms and its harmonics. This showed the necessity of combining multi-lead features to classify different AF mechanisms.

#### Evaluation of classifiers

We evaluated the classification results of our features derived by SO-BSS with the following AF-complexity metrics in Table 2. The first metric was AFFTr_2*DF*_, which has been used to distinguish between FS and reentrant sources [28], computed as the sum of normalized FFT power spectrum density of 0–2 × DF of the V1-lead ECG signal. The second metric was the nondipolar component index (NDI), which was used to predict the acute ablation outcome in persistent AF patients from BSPMs [29], measured as the proportion of the residuals outside of the three principal axes. We replaced the SO-BSS features with these metrics, and reused the same classification pipeline for training and testing, i.e. SMOTE and random forest classifier.

**Table 2:** Testing scores using nested leave-one-patient-out cross validation on all classification tasks over five patients, using features of SO-BSS, AFFTr_2*DF*_ and NDI. Bold fonts mark the highest scores and the corresponding features.

Task	Feature (with signal used)	Accuracy (mean±s.d.%)	Precision (mean±s.d.%)	Recall (mean±s.d.%)
FS presence	**SO-BSS (BSPM)**	**90.6±1.0**	**96.8±0.7**	**89.7±1.2**
	**SO-BSS (ECG)**	**90.6±0.6**	**97.2±0.7**	**89.4±1.2**
	AFFTr_2*DF*_ (V1)	56.8±2.2	73.2±1.9	61.3±2.7
	NDI (BSPM)	60.8±2.3	75.9±1.7	65.2±3.6
AF sustainability	**SO-BSS (BSPM)**	**93.1±0.6**	**90.4±3.0**	**93.0±3.1**
	**SO-BSS (ECG)**	**94.2±1.2**	**92.4±2.6**	**93.4±3.3**
	AFFTr_2*DF*_ (V1)	58.5±2.0	48.2±3.7	54.1±4.0
	NDI (BSPM)	61.6±2.3	52.1±5.1	57.4±5.7
AF sustainability (evaluated on FS-driven episodes)	**SO-BSS (BSPM)**	**91.5±1.2**	**69.6±6.8**	**83.0±11.6**
	**SO-BSS (ECG)**	**92.7±1.8**	**74.7±6.3**	**82.4±13.1**
	AFFTr_2*DF*_ (V1)	60.3±3.0	20.5±2.6	55.7±4.8
	NDI (BSPM)	63.2±3.0	22.6±4.2	56.5±4.6
FS location	**SO-BSS (BSPM)**	**80.0±6.6**	**81.2±9.0**	**75.0±8.4**
	SO-BSS (ECG)	61.0±5.2	59.3±6.8	53.6±8.4

On FS presence and AF sustainability, our method achieved high scores of accuracy, precision and recall, all close to or above 90% with variance ≤ 3.3%, significantly outperforming AFFTr_2*DF*_ and NDI, showing the efficacy of our algorithm, as well as robustness to inter-patient variability in atrial geometries. We also evaluated the performance of the AF sustainability classifier on FS-driven episodes, in order to show the predictability of AF sustainability after the removal of FS on patients known to experience atrial ectopic activities. When the AF sustainability classifier was evaluated on FS-driven episodes, the accuracy was still high, but the precision and recall scores slightly decreased. This showed that AF sustainability classifier can be effectively predicted from FS-driven episodes, rather than only separating between FS-only or reentry-only episodes, which is a much more trivial task. These scores were mainly impacted by FS with a CL of 210 ms, as shown in S2 Table, which was expected given that the initiator-type FS had a different mechanism at CL of 210 ms as discussed in the previous section, and AF inducibility at this CL also presented a considerably larger inter-patient variation (12.5%) compared to other CLs (≤ 3.2%), as shown in the third column of S2 Table. Although we did not apply any specific location information of the atria nor the torso leads, the estimation of focal site region achieved moderately high accuracy of 80.0% and 61.0% from BSPMs and ECGs.

**Fig 5:**
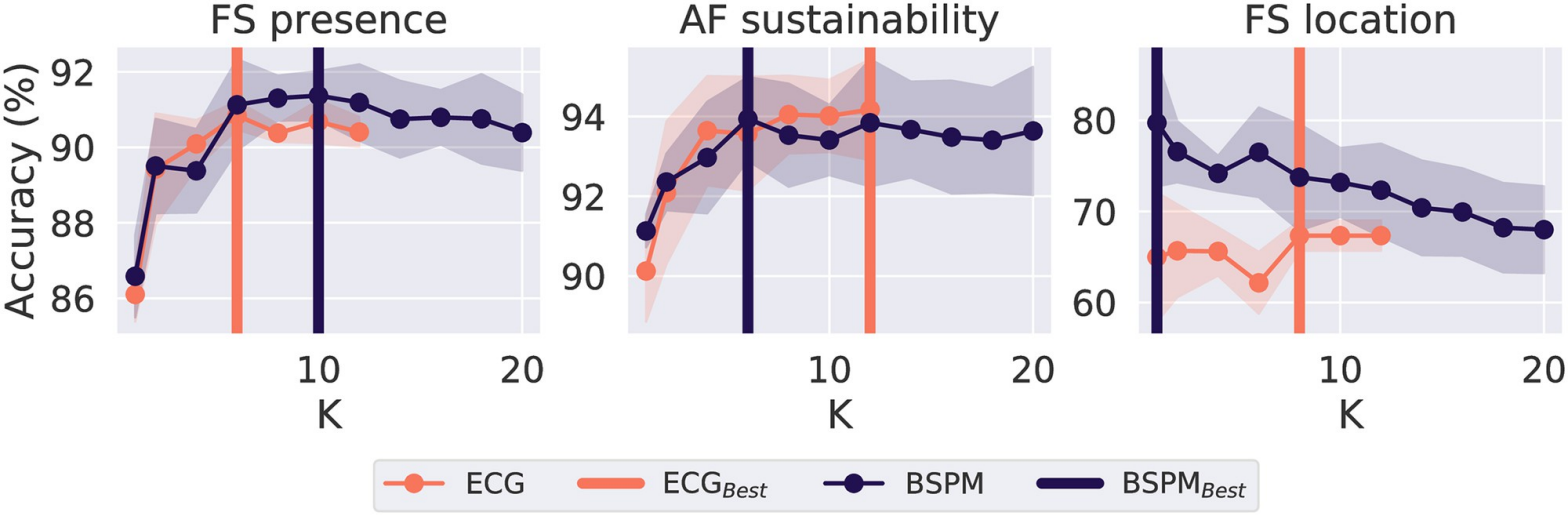
Means (points) and standard deviations (shaded areas) of the test accuracy scores using leave-one-patient-out cross validation, for changing *K* and lead systems (ECGs or BSPMs), on the classification tasks, with the best *K* for the highest accuracy (ECG_*Best*_ or BSPM_*Best*_) in each setting shown by vertical bars.

To understand how different mapping systems and the number of sources *K* as the input parameter of SO-BSS affect the classification performance, we calculated the mean and standard deviation of the accuracy score across all five outer-validation sets in Fig 5. Both FS presence and AF sustainability classifiers presented similar trends, where the accuracy increased up to a certain *K* (*K* = 6) with little change afterwards, likely due to the inherently small number of sources with distinctive CLs. For the FS location classifier, with a larger *K*, the accuracy dropped for BSPMs but increased for ECGs.

**Fig 6:**
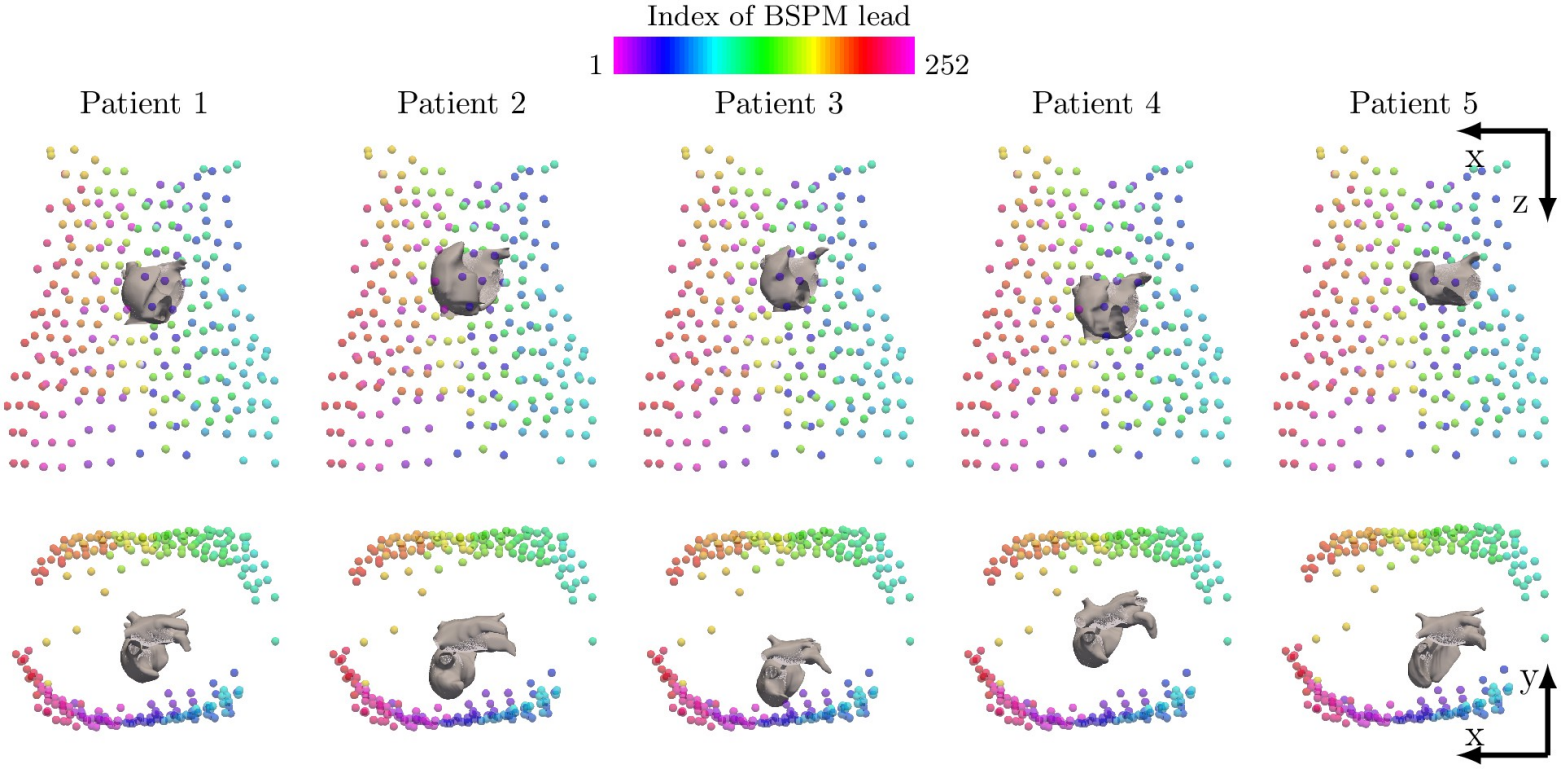
Meshes of five patient atria, and locations of 252 vest leads to compute BSPMs. Each column shows the data for one patient. The top row shows the front view and the bottom row shows the top view. Inter-patient variations of atrial shapes and positions are visible.

To test the sensitivity to different vest placements, we varied lead positions by 10 and 20 degrees around the z axis via the center of mass, and 5 and 10 cm in the x, y, and z directions to test over the dataset of Patient 2, using *K* = 10 sources for SO-BSS with BSPMs as shown in Fig 6. These variations are considered extreme cases for intra-patient variations, only designed to test the robustness of our algorithm. The results of the sensitivity tests for the FS presence and AF sustainability classifiers and the dominant CL estimation are shown in S3 Table. The absolute differences of the estimated dominant CLs between all vest variations and the original vest placement were small, with mean ≤ 7.09 ms and standard deviation ≤ 30.0 ms. For FS presence and AF sustainability classifiers, all vest placements received similar classification scores with respect to each other, with the maximal absolute difference being 9.6%. In terms of the FS location classifier, the classification scores remained similar only for rotation of -20 to 10 degrees around the z axis and -5 cm translation in the y direction (maximal absolute difference ≤ 10%), but other changes by translation resulted in larger differences. This is expected, as the **s**_1_-to-leads contribution used in this classifier is a spatial feature (assuming fixed locations of electrodes), which is by design sensitive to large variation of lead placements.

#### Feature interpretation of classification

**Fig 7:**
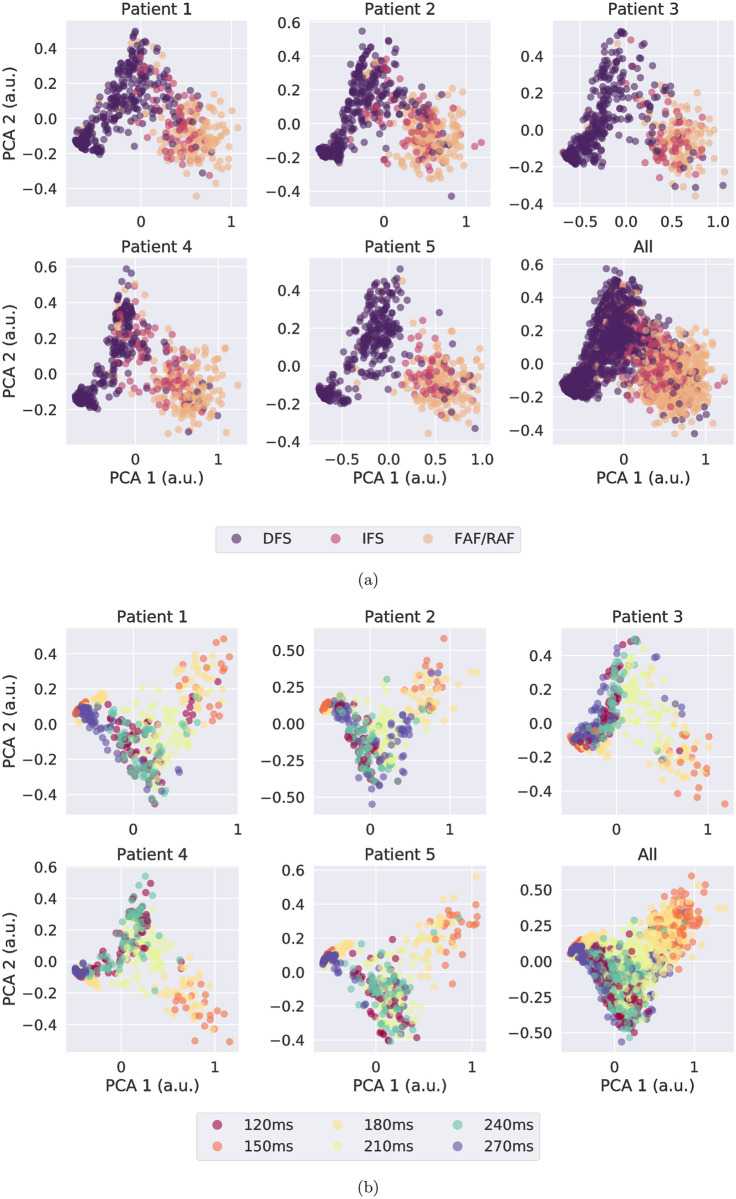
Two-dimensional PCA representation of MaxAC values obtained from SO-BSS with *K* = 10 over simulated BSPMs), on each patient and all pooled, for different colour-coded groupings: (a) all categories over all episodes, and (b) focal CLs over all FS episodes. Clustering based on categories and focal CLs can be seen for each patient. a.u.: arbitrary unit.

The MaxAC values, as part of the feature vector, encoded the information about multi-source periodicity as signatures of atrial events. The MaxAC values of sources extracted by SO-BSS with *K* = 10 from BSPMs were visualized by being projected onto two-dimensional orthogonal bases using Principal Component Analysis (PCA) [30], colored by their ground-truth mechanisms or CLs, as shown in Fig 7a and 7b, respectively. The PCA captured variances 93.3±0.6% and 93.2±0.6% in the reduced dimensions. On all five patients, the driver-type FS could be easily separated from the other mechanisms, but there was a more complex decision boundary between initiator-type FS and driver-type FS. CL clusters could also be seen in the feature space, such as CL clusters of 150 ms and 180 ms, both of which were AF-susceptible CLs, were adjacent to each other in all patients.

SO-BSS were able to capture the FS in most of the cases. This can be seen in S8 Fig that the estimated dominant CL, extracted by SO-BSS with *K* = 10 from BSPMs, well approximated the ground-truth focal CL of the FS or its harmonics. The misalignment between the dominant CL and focal CL was mostly caused by the presence of reentrant activities during initiator-type FS. For some driver-type FS with a CL of 150 ms, the dominant CL was estimated to be around 225 ms, which is the average of focal CLs of 150 ms and 300 ms, the latter resulting from 2:1 conduction blocks. On 98.1% FS, their focal CL was estimated by at least one source or their harmonics, with an absolute error being ≤ 5 ms. The focal ablation increased the dominant CL of initiator-type FS (means of 195 ms (before) and 204 ms (after), p-value < 0.0001, pairwise one-sided t-test).

### Applying trained classifiers to predict patient’s AF mechanisms

Having seen good classification results on our simulated data, the next step was to apply our trained classifiers on the clinical dataset of 52 paroxysmal AF patients, in order to test if they bring in any predictive value for the outcome of ablation treatment. We used the classifiers trained on the BSPMs of the virtual cohort, with *K* = 10 for FS presence and AF sustainability and with *K* = 1 for FS location, to predict signal-level mechanisms from patient BSPMs. We then obtained the patient-level mechanisms by aggregating all signal-level mechanisms seen in at least one signals of the patient. The number of patients with each patient-level AF mechanism is shown in Table 3. All patients had an arrhythmogenic substrate. A patient was counted as Group 1 if initiator-type FS were found in one or more signals, and all of them originated from the same atrium. Initiator-type FS were found in more than 70% of patients.

**Table 3:** Groupings and counts of patient-level mechanisms (by row), as well as the assignment of the patient group for survival analysis in Fig 8, based on whether a patient contains initiator-type FS coming from a single atrium. This table omits the results of patients with driver-type FS for clarity.

Mechanism	Initiator-type FS		Sustained AF without FS	Count
	from LA/PVs	from RA		
Group 1 (Single-atrial initiator-type FS)	✔		✔	13 (25%)
		✔	✔	14 (27%)
Group 2 (Other mechanisms)	✔	✔	✔	11 (21%)
			✔	14 (27%)
Count	24 (46%)	25 (48%)	52 (100%)	52 (100%)

**Fig 8:**
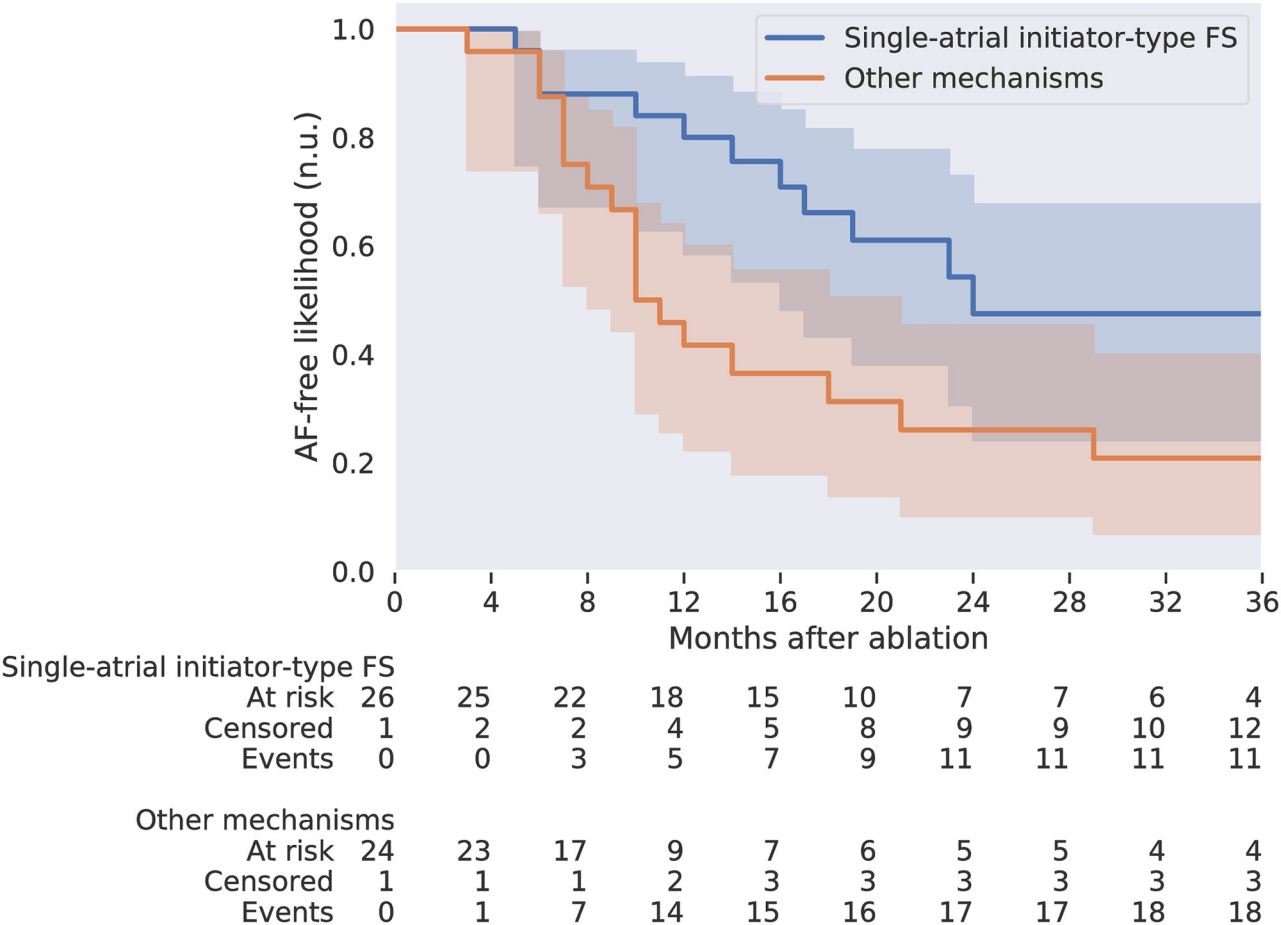
Kaplan–Meier curves of two paroxysmal AF patient groups, showing the post-ablation AF-free likelihoods up to three years. The grouping was according to whether the AF episodes in the patient were predicted as driven by initiator-type FS originating from a single atrium. The shading shows the 95% confidence interval of the Kaplan–Meier curves. The first group had better two-year and three-year AF-free outcomes than the other group, with p-values of logrank tests both < 0.01. n.u.: normalized unit.

The simplest AF mechanism was driven by an FS from a single atrium, which was also the most straightforward driver to map and ablate during a catheter ablation procedure. Therefore, we divided the patient cohort according to whether each patient contained an initiator-type FS originating from a single atrium. We calculated 36-month Kaplan–Meier curves [31], and logrank tests on the difference of the two Kaplan–Meier curves, up to one-year, two-year and three-year follow-ups, using the lifelines Python package (version 0.26.0) [32], as shown in Fig 8. A higher post-ablation AF-free likelihood was observed for the patient subgroup with initiator-type FS originating from a single atrium than the other group, with p-values < 0.01 for both two-year and three-year follow-ups, and p-value > 0.05 for one-year follow-up. This demonstrates that by classifying patient AF mechanisms using their pre-operative BSPMs, we successfully predicted a patient subgroup effectively targeted by the current procedure.

## Discussion

In this study, we developed three unique binary classifiers using non-invasive signals, with which we can inform AF ablation targeting. Specifically, we detected the presence and the location of FS, as well as the arrhythmogenesis of substrate for sustained AF. We used highly detailed multi-scale computer models of the atria which not only generated signals for a cohort of patients, but also, importantly, allowed us to interpret the biophysical basis of the signals. On our cohort of 52 paroxysmal AF patients, our classifiers demonstrated their ability to identify patients who are more likely to have sustained benefits from current ablation techniques.

### Clinical values

To the best of our knowledge, our method is the first to non-invasively detect FS and arrhythmogenic substrate from body surface potentials to suggest AF ablation targets. Our approach non-invasively determines where the AF condition of a patient lies on the AF spectrum, with drivers ranging from local FS to more diffused multiple wavelets [10], which can advance current AF treatment towards mechanism-targeted therapy.

We demonstrated the prognostic value of our classifiers using pre-operative BSPMs of 52 paroxysmal AF patients. Predicted by our algorithm, more than 70% of the patients had an initiator-type FS, consistent with our above-mentioned in-silico finding and clinical observations. The ablation procedure was the most effective for patients with AF episodes driven by initiator-type FS originating from a single atrium, shown by their improved two-to-three-year AF-free rates, demonstrating the usefulness of our method in predicting the long-term ablation success for patients going through AF ablation, using only their pre-operative BSPMs. This marked a successful prototype of non-invasive per-operative screening for mechanism-directed treatment. Note that our method is not limited to the demonstrated procedure, as it could predict the success of a given treatment by determining whether the patient’s AF mechanism is by design targeted by that treatment.

Our classifiers could also provide guidance during an ablation procedure. Consider an AF patient going through ablation: from body surface potentials, either ECGs or BSPMs, our method identifies an FS on the LA with a substrate that perpetuates AF. After isolating the focal site, if the AF does not terminate, patient body surface potentials are again analyzed by our method, to detect additional FS and its arrhythmogenesis, in order to assess the necessity and strategy for further intervention. This process continues until the condition for the ablation end-point is met, such as no more FS remains, or the AF substrate is altered. The iterative manner of our method makes it also applicable to atria with multiple foci, given a single FS dominates an episode, and results in minimal ablation lesions and thus reduces complications. The non-invasive localization of FS could also save time in mapping. In addition, the non-invasive manner, the fast execution, and the adaptability into an existing workflow without human input, makes it ideal for a real-time mapping tool to support clinical decisions.

Another use of our method can be found for health management in the general population especially in aged groups. A positive result of AF sustainability along with a presence of FS indicates malignant FS which leads to sustained AF. This compliments the current ECG analysis using only ectopic beat counts to predict AF incidents in health checkup of the general population [4–6].

### Sustained rotors during FS-driven episodes suggest an arrhythmogenic substrate

An important observation we made was that the AF sustainability after removing the FS, or the AF inducibility of the FS, was correlated with the presence of sustained rotors when the FS was active. This is based on the observation that initiator-type FS were predominantly rotor-driven (66.7%), a much higher proportion than driver-type FS (2.6%), with a p-value < 0.0001. There was also a high AUROC score of 0.83 between the rotor duration and the AF inducibility. This observation provides a mechanistic basis for predicting the AF inducibility after the focal ablation using pre-ablation signals.

### Factors that impact AF inducibility from FS

The AF inducibility from FS varied under different pacing and substrate conditions, as can be seen in Fig 2. The variability in critical CL range for AF inducibility can be explained by tissue properties, described by refractorness and spatial APD heterogeneity.

The wavelength, which measures the travel distance of wavefronts within the ERP, is believed to be critical for inducing self-sustained AF [33]. Under the model of wavelength, a shorter ERP would thereby shorten the critical CL of the rapid pacing in inducing AF. Combining Figs 2 and 4, it became clear that shorter AF-susceptible CLs in the LA were correlated with the shorter ERP in LA. The critical CL range of AF inducibility in the LA was also decreased from 180 ms to 150 ms by ACh regulation, due to APD shortening. Furthermore, from our model, the focal CL of 180 ms was associated with the highest AF inducibility, which was very close to the average focal CL of 175 ms observed in AF patients [1]. This evidence showed that our models were representative of AF patient population.

The spatial APD heterogeneity was another important factor, which provided an important substrate for both formation and maintenance of rotors. Because of the spatial APD heterogeneity and the conduction slowing at the LA/PV junction [34], AF inducibility was the highest for FS on the PVs followed by the LA in our results, which also agrees with non-invasive mapping results of Haïssaguerre and Rudy [14, 16, 35] and intracardiac rotor mapping results [36]. ACh regulation further contributed to the spatial APD heterogeneity (Fig 4), consistent with other modelling studies [37, 38]. Little difference in AF inducibility on the RA foci was found by adding ACh on the LA, which shows that it is the proximity to APD heterogeneous region that promotes AF inducibility.

### Mechanism-inspired classifiers

The analysis above suggested our simulations were reasonably representative of AF observed in patients, and thus, were ready to be used as the training data for non-invasive classifiers. It also established that AF sustainability could be predicted from FS-driven episodes by two main features, focal CL and the presence of sustained rotors. These features are, however, usually extracted from invasively-acquired intra-cardiac electrograms. To translate these features to non-invasive measurements, in order to detect the FS and the arrhythmogenic substrate, we approximated them with atrial sources extracted by SO-BSS from body surface potentials. As a result, the FS and the reentrant sources can be separated even in the case of co-existence, such as in the case of initiator-type FS, which demonstrated usefulness in AF ablation targeting.

The focal CL was well approximated by the dominant CL, extracted by SO-BSS for the driver-type FS and most of the initiator-type FS (S8 Fig). It was more advantageous to use the multi-lead SO-BSS than ACF or FFT analysis on the V1 lead, as SO-BSS extracts a spectrum of CLs. In addition, on one-second 1 kHz body surface signals, the resolution of FFT-extracted DF was 1 Hz, much lower than the resolution of 1 ms in our method. For the initiator-type FS which contained both FS and reentrant sources, their dominant CL increased after the removal of FS. This was consistent with the gradual increase in the CL with more extensive ablation steps [39]. Such an observation strengthens faith in our model, and shows that our predictor could distinguish FS in spite of the presence of re-entrant sources.

The presence of sustained rotors was captured in the MaxAC features. On a PCA-reduced space of all MaxAC features, a clear boundary can be seen between driver-type FS and other types, showing a distinctive difference between MaxAC features with and without an arrhythmogenic substrate, respectively (Fig 7a). The CL clusters in the MaxAC feature space (Fig 7b) also show that the MaxAC features encoded information about the focal CL, via their representation of atrial states, i.e. reentrant sources, FS, or both.

By using the contribution from the first periodic source to each lead (**s**_1_-to-leads) during FS-driven episodes, we were able to predict whether the FS originated from the LA or the RA. This feature was again inspired by the periodic nature of FS. An advantage of using BSPMs in inferring spatial information, compared to ECGs, was also proven by higher classification scores with BSPMs.

Our random forest classifier automatically combined the aforementioned heterogeneous features without additional re-scaling for classification. By doing so, a complex decision boundary between classification target classes was formed, whilst reducing overfitting by assembling the results between multiple decision trees.

### Robustness of classifiers

Our methods achieved high accuracy, precision and recall scores in Table 2, outperforming the AFFTr_2*DF*_ [28] and NDI [29] features. We highlight that our non-invasive classifiers were robust to inter-patient variability, given a large variation of the atrial positions and shapes (Fig 6). In particular, the classification of FS presence and AF sustainability, as well as the estimation of the dominant CL, were robust to large changes to vest placement (up to 10 cm in translation and 20 degree in orientation as shown in S3 Table) that were not represented in the training dataset. This was because the heart-torso variability was digested by the transformation matrix during source reconstruction of SO-BSS, and neither the features of these two classifiers nor the dominant CL relied upon the transformation matrix. For other technologies however, such as ECGi, inferred atrial potentials from BSPMs could be quite sensitive to the relative positioning and orientation of the heart within the torso [17, 40].

Compared to ECGs, the usage of BSPMs yielded a better performance in classifying the FS location, because the additional leads of BSPMs provided a more detailed view of the **s**_1_-to-leads contribution. On the other hand, using BSPMs and ECGs led to similar classification results on the other tasks (“FS presence” and “AF sustainability”), which shows that 12-lead ECG signals were sufficient in extracting the *K* common periodic sources needed for these tasks. The joint diagonalization is robust to white noise, as there is no common structure of second-order statistics in it. Therefore, our algorithm is resilient to white noise.

Our FS presence and AF sustainability classifiers were insensitive to the number of sources for SO-BSS when *K* is larger than a certain number which is about 6 (Fig 5). The robustness of changing *K* means that a prior knowledge about atrial sources is not required, and a smaller number of sources (< 6) can be selected for faster computation with a moderate accuracy. To detect FS and AF sustainability, *K* = 10 sources are recommended with either ECGs or BSPMs, and *K* = 1 with BSPMs is recommended for predicting the focal site region.

Furthermore, our method does not require imaging the heart nor the torso, which accelerates the mapping process, and makes the tools accessible for both clinical and personal usage.

### Related studies on AF inducibility from FS

Given the same pacing duration, only some FS with certain CLs and tissue conditions managed to induce self-sustained AF, suggesting that using the ectopic beat counts to predict for AF incidents in previous work [4–6] could be improved by including a prediction of AF sustainability by our work.

Gong *et al*. [41] also showed that both focal timings and locations were essential to AF inducibility of FS, with the spatial dispersion of APD as an underlying mechanism. We reduced one of the timing factors, the coupling interval conduction between the sinus nodal stimulus and FS, by excluding the sinus rhythm from the experiments. This was based on the assumption that the low-frequency sinus rhythm plays a limited role in affecting the higher-frequency FS [42]. The consistency with previous findings suggested that our generated episodes represented patient AFs faithfully. The smaller search space also reduced the required number of simulations needed for training robust prediction models. Moreover, we added ACh regulation in the experiments which further diversifies our synthetic dataset in studying factors that impact the AF inducibility from FS.

The AFFTr_2*DF*_ feature was also developed on simulated episodes, but was only tested on FS of 3 Hz and 5 Hz. This rarely induced complex patterns such as rotors or 2:1 atrial responses that were commonly observed in patients, confirmed by the morphology of FS in their results. In addition, their calculated power density spectrum, calculated from 500 ms 500 Hz signals, had a resolution of 0.01 Hz, much higher than the theoretical frequency resolution of FFT, which we were not able to reproduce. In comparison, our work not only considered a wide range of focal CL in FS, some of which co-existed with reentrant sources, but also added ACh regulation to further increase the spatial APD heterogeneity of the LA. Therefore, our classifiers, being trained and evaluated on more realistic simulations of FS and reentrant sources, were more robust.

The NDI feature, as an AF complexity metric already applied on AF patient signals, did not perform well on our tasks either, with a performance similar to the AFFTr_2*DF*_ feature. This may be due to the fact that their defined organized sources, captured by the three principal axes of PCA, could still include some organized reentrant sources.

Another simulation study [43] also presented a classifier to detect PV versus non-PV drivers with the 12-lead ECG, which successfully predicted the acute success of PV isolation in 46 AF patients. Our AF sustainability classifier does not restrict the target procedure to PV isolation, and more importantly, detects an arrhythmogenic substrate that indicates the AF susceptibility in the face of new AF triggers. This is more appropriate for predicting mid-term and long-term outcomes of a given treatment.

### Limitations

The same model parameters were used across different meshes. We assumed our targeted paroxysmal AF patients were screened at an early stage of AF, so we did not add scar or fibrosis to our model. We simulated the focal ablation by stopping the focal discharges, without introducing ablation lesions. We approximated body surface potentials with extra-cellular potentials on the vest electrodes, without inscribing the conductivity properties from the heart to the torso surface. These limitations could be removed in future work.

The other type of initiator-type FS initiated macro-reentry, which occurred much less frequently than driver-type FS. The similar AF inducibility from FS with a CL of 210 ms across all focal site regions, alongside with a large inter-patient variability in the AF inducibility on this focal CL, suggests that global geometrical factors shared by both atrial chambers, may need to be considered for predicting AF inducibility from FS.

The follow-up records for paroxysmal AF patients contained missing entries, and were retrospectively interpreted.

## Materials and methods

### Ethics statement

This study has been approved by the institutional review board of the Haut-Lévêque Cardiology Hospital (Bordeaux, France), and written consent was previously obtained from all patients.

### Method overview

Our methods are summarized in the Step 1 to 4 of Fig 1. We first present the generation of driver-type FS, initiator-type FS, FS-induced AF and reentrant AF episodes for our synthetic AF dataset, for which their ECGs and BSPMs were also computed. We then show the atrial source reconstruction using SO-BSS, from which features for the random forest classifiers were extracted for non-invasive prediction. Finally, we introduce our paroxysmal AF patient dataset, and present the application of the trained classifier to predict AF mechanisms of patients.

### Construction of the synthetic AF dataset

To construct the synthetic AF dataset for training the classifiers, we first modelled AF episodes driven by FS and reentrant drivers, and applied focal ablation.

Patient bi-atrial meshes were made from late-gadolinium enhanced magnetic resonance imaging scans of five AF patients from the Haut-Lévêque Cardiology Hospital (Bordeaux, France) [34, 44]. Their orientations were aligned, and their centers of mass had standard deviations of 1.3, 1.8 and 1.0 cm in the x, y, z directions, respectively. The meshes made from computerized tomography imaging of the atria and 252-lead vest (CardioInsight, Medtronic, MN) from a sixth patient were also exported. The atrial mesh of the first patient was registered with that of the sixth patient by matching the centers of mass of the two atria by linear translation. All five virtual patients then shared the same vest leads seen in Fig 6. Meshes had on average 489k nodes, and 1.18M elements, primarily triangular elements, with an average edge length of 318 *μ*m (S4 Table). The average edge length of 350 *μ*m in triangular elements was shown to produce realistic reentrant waves [45] while reducing computational cost. In addition, as shown in previous studies [34, 44], clinically observed behaviour, such as the density and frequency of rotors near pulmonary veins and in fibrotic regions, is well captured with this choice of simulation parameters. The transmural conduction was modeled by the linear elements between the endocardial and epicardial layers as in Labarthe *et al*. [46]. Inter-atrial structures were added to the meshes [46] and additionally, an isolated sinoatrial node was implemented [42]. To specify locations of non-excitable regions and foci of all five meshes at comparable sites between different subjects in a common coordinate system, we used the universal atrial coordinate system [47], computing *α*_*LA*_, *β*_*LA*_, *α*_*RA*_, and *β*_*RA*_ to describe the location of each vertex.

All episodes of FS and AF were simulated using the CARPentry software [48]. Based on the previous works [34, 44], heterogeneous ionic model parameters and fiber orientations were assigned to different regions of the atria, and the conduction slowing at the LA/PV junction was manually created. We modified the LA and its appendage, where a human non-fibrotic ACh-regulated AF ionic model [38] was used. In order to match the conduction velocity slowing of the LA and LA appendage for AF patients [38], we scaled the intercellular conductivities of the crista terminalis, pectinate muscle, Bachmann’s bundle, coronary sinus and RA myocardium by a factor of 0.336, which is the geometric mean of the reduction for AF models in the conductivities in the longitudinal and transverse directions to the fiber compared to non-AF models [38]. To add APD heterogeneity to the model, we also simulated islands of ACh release ([ACh] = 0.1*μ*M) on the LA surface and appendage [38], where 60, 80 and 100 2mm-radius ACh islands were randomly distributed at areas of *α*_*LA*_ of 0–0.33, 0.33–0.67 and 0.67–1 and *β*_*LA*_ of 0–1, respectively. An example ACh distribution is shown in S9 Fig. Both FS and reentrant AF sources were applied in conditions with ([ACh] = 0.1*μ*M) and without ACh.

Following the original bi-layer model developed by Labarthe *et al*. [46] as well as the subsequent studies on AF properties built on the bi-layer models [34, 44, 49], we ran monodomain simulations using a Crank-Nicolson scheme as the time integrator with a time resolution of 20 *μs* for diffusion of current within the tissue. This protects more against elements with poor aspect ratios than improves accuracy, since the CFL condition is stricter with forward Euler. Within the ionic cell model, depending on the stiffness of the particular state variable, a combination of Euler forward, Rush-Larson or backward Euler may be used. Furthermore, nonuniform integrators like CVODE, may be further employed for subsets within ionic models with output sampled at 20 *μs*. The time resolution of 20 *μs* is fine enough to capture the propagation of the fastest sodium current. Additionally, changing the time resolution from 10 to 50 *μs* did not impact the propagation speed, regardless of the spatial resolution [50]. As shown in previous studies [34, 44, 49], clinically observed behaviour is well captured with this choice of simulation parameters. Extracellular potentials were computed from transmembrane potentials using a classical *ϕ*_*e*_ recovery method [51] at the 252 lead points of the vest to constitute the BSPM. The ECG was calculated from a subset of the BSPM potentials. To recreate clinical recordings, potentials were referenced to the Wilson Central Terminal.

#### Simulations of FS and focal ablation

Single-site repetitive pacing at specified locations was performed with CLs of 120, 150, …, 270 ms to model the clinically observed range of focal CLs [1], over a period of 3000 ms to reach a stable state for the FS. Focal sites were located over the myocardial sleeves of the four PVs as well as at 32 locations of the LA and the RA at combinations of (*α*, *β*) with *α*_*X*_, *β*_*X*_ ∈ {0.2, 0.4, 0.6, 0.8} and *X* ∈ {*RA*, *LA*}. Each FS is identified by its focal site, CL, as well as [ACh].

We tested whether each FS induced self-sustained AF lasting for 1000 ms after stopping the FS, to mimic focal ablation. If AF stopped after focal ablation, the FS was the driver and denoted as a driver-type FS. Otherwise, the FS induced AF, and was denoted as an initiator-type FS. The 1000 ms AF segment after the pacing stopped for an initiator-type FS was labelled as FS-induced AF. The last 1000 ms segments of constant FS pacing, as well as the 1000 ms segments of FS-induced AF after pacing stopped, were added to our synthetic dataset.

#### Simulations of reentrant AF

To initiate reentrant AF, we used a phase distribution method described in an AF simulation study [52]. We added a non-excitable core region by setting the tissue conductivity to zero, with radii of the region being 0 cm, 0.5 cm or 1 cm at the same 32 sites as the focal sites on the atria, to vary the initial conditions for reentrant sources. The atrial surface was divided into 48 equal sectors around the core to determine the initial conditions for each sector. The states for the sectors were taken from 48 time instances equally sampled from an action potential after a complete depolarization, simulated by the same ionic model with the located atrial surface. A demonstration can be found in S10 Fig.

Only those simulations that sustained for more than 3000 ms were kept for further investigation, and the last 1000 ms segment of the resultant AF was marked as reentrant AF, and added to our synthetic dataset. This interval of 3000 ms was adopted so that their dynamics are regular, comparable to FS. Additionally, a rotor lasting for more than 3000 ms is also likely to be sustained for a long time, say, 80 seconds [53].

#### Data analysis

In total, there were 5 (patients) ×36 (LA, RA and PV foci) ×6 (CLs) ×2 ([ACh]) = 2160 FS cases, and a small number (60) of them were not launched as their universal atrial coordinates of foci referenced locations outside of the atrial tissue, as some coordinates pointed to cavities such as those of PVs. This left 2100 FS simulations, with 900 LA, 960 RA and 240 PV FS. The odds of inducing self-sustained AF for at least 1000 ms from FS under a specific condition, measured as the proportion of initiator-type FS, was termed *AF inducibility*. The AF inducibility test identified 1776 driver-type FS, and 324 initiator-type FS, which entailed another 1776 discarded simulations and 324 FS-induced AF simulations. We initiated 5 (patients) ×32 (LA and RA rotor core sites) ×3 (core radii) ×2 ([ACh]) = 960 reentrant AF simulations, with 549 sustaining for more than 3000 ms being preserved. These 1776 (driver-type FS) + 324 (initiator-type FS) + 324 (FS-induced AF) + 549 (reentrant AF) = 2973 segments were used as study data in the following steps.

In order to track rotors in these simulations, we calculated phase singularity points, i.e., the cores of the rotors. We first filtered the transmembrane voltage with a fourth-order 15 Hz low-pass Butterworth filter, removed the DC component with a 0 Hz notch filter, computed phase by applying the Hilbert transform, and then applied the Iyer-Gray method [54] which identifies phase singularity points as those around which a contour integral of the phase equals ±2*π*. To calculate sustained rotors, we kept rotors lasting for at least 100 ms, assuming the phase singularity point moves less than 2 mm within 1 ms. The total duration with at least one such rotor was also computed for analysis.

Local activation and repolarization times were marked by the upward-sloped intersection of -30 mV and downward-sloped intersection of -70 mV of the transmembrane voltage since the time of a focal discharge, respectively. We then calculated the APD by subtracting the repolarization time from the local activation time. ERP was estimated as the maximal focal CL whereby the FS encountered a complete conduction block at tissue surrounding the focus. Searched on the set of prescribed focal CLs, ERP was estimated between the maximal CL and the minimal CL where a complete conduction block did or did not occur around the pacing site, respectively.

The proportional z-test was implemented by statsmodels Python package (version 0.11.1) [55]. The student t-test was implemented by pingouin (version 0.3.10) [56].

### Non-invasive classifiers

#### Equivalent atrial source extraction with SO-BSS

We first extracted *K* equivalent periodic atrial sources (**s**_1_, **s**_2_, …, **s**_*K*_) and their CLs (CL_1_, CL_2_, …, CL_*K*_) from the body surface potentials, ranked by the source periodicity, using a SO-BSS method adapted from SOBI [57]. The implementation details can be found in S1 Appendix.

The periodicity of the extracted source over a time-lag *τ* was measured by an unbiased version of auto-correlation function (ACF), an empirical periodicity measurement for time series **s**(*t*) as a function of a time-lag *τ* [58]:

$$\begin{array}{c} \text{ACF}\left(\tau \right)=\frac{T-1}{T-\tau -1}\frac{\sum_{t=1}^{T-\tau }\left(s\left(t\right)-\bar{s}\right)\left(s\left(t+\tau \right)-\bar{s}\right)}{\sum_{t=1}^{T}{\left(s\left(t\right)-\bar{s}\right)}^{2}} \end{array}$$

with $\bar{s}$ being the expected value of **s**(*t*).

The estimated CL of each source **s**_*i*_ was adopted from the time-lag within a set of time-lags *T*_*FS*_ (see S1 Appendix for selection of *T*_*FS*_) resulting in the maximal auto-correlation score of the source **s**_*i*_,

$$\begin{array}{cc} {\text{CL}}_{i} & =\underset{\tau }{\text{argmax}}\;{\text{ACF}}_{i}\left(\tau \right),\tau \in T_{FS}\\ {\text{MaxAC}}_{i} & ={\text{ACF}}_{i}\left({\text{CL}}_{i}\right) \end{array}$$

To approximate the dominant rhythm in the atria, a dominant CL was taken by the first CL_*k*_ from *k* = 1, 2, …, *K* that satisfied ACF_*k*_(CL_*k*_) > CI_*k*_(CL_*k*_), where CI_*k*_(*τ*) denotes the range of 95% confidence interval for estimating ACF_*k*_(*τ*).

On a Intel Xeon Gold 6140 processor with 36 cores, the time to extract SO-BSS features on a 1000ms BSPM signal sampled at 1 kHz was around 0.2 second with *K* = 1, around 0.3 second with *K* = 10, and around 2.4 seconds with *K* = 20.

#### Classification

Three binary classifiers were formed: (1) *FS presence*, comprised of the union of initiator-type FS and driver-type FS as the positive class, and the rest being the negative; (2) *AF sustainability*, to predict if there is an arrhythmogenic substrate where AF can be sustained in the atria without focal discharges from the FS, where the positive class consists of the union of FS-induced AF, reentrant AF and initiator-type FS, and the negative class is driver-type FS; (3) *FS location*, trained and evaluated on the FS-driven episodes, to determine if the focal site is located on the RA (positive class), or from the LA or PVs (negative class). We also evaluated *AF sustainability (with FS)*, which used the trained classifier from *AF sustainability* to distinguish between initiator-type FS (positive class) and driver-type FS (negative class).

An illustration of features for each classifier is shown in Fig 1 Step 2. The classification of FS presence and AF sustainability requires a combination of multiple periodic sources to effectively represent AF. To encode the sources in a vector representation, we used two characteristics, CLs and MaxACs, to represent each source, as the ACF of most extracted sources, especially those with a high periodicity, resembled sine waves with different periods. For *K* extracted atrial sources, 2 × *K*-dimensional characteristic features were used as input features for classification.

The features of the FS location classifier were the **s**_1_-to-leads contributions, specifying the contribution of the highest ranked periodic source **s**_1_ to the normalized signal of each lead. This encoded the spatial information of the first periodic source. As the SO-BSS sources are sign-agnostic, the signs of coefficients were fixed to one where a majority of coefficients were positive. The coefficients of all **s**_1_-to-leads vector were re-scaled to have the same maximum and minimum.

The imbalanced sample sizes of each class may result in a classifier biased towards the majority class. To overcome this problem, we applied an oversampling algorithm SMOTE [59] to make sure that all classes had the same number of training examples. This was then followed by a random forest classifier [24], which fits a non-linear boundary between the two classes, as shown in Fig 1 Step 3. It achieves this by splitting the parameter space of each feature in a way that maximizes the information gain. We fixed the number of decision trees in the random forest model at 200.

To train and evaluate a model on the five simulated patient datasets, we adopted a nested leave-one-patient-out cross validation method, which reduced bias in estimating the true error of the classifier on small datasets [60]. There were two nested cross-validation loops, where the outer loop split the training/validation set and the one-patient test set. In the inner loop, the classifier was trained on the training set, and selected the best hyperparameter resulting from the highest classification accuracy on the one-patient validation set. A model was then retrained with the best hyperparameter on the entire training/validation set, and evaluated on the test set as the performance of the classifier. The hyperparameter to select was the number of SO-BSS sources, *K*.

The SO-BSS algorithm was implemented in Python (version 3.6). SMOTE was implemented by imbalanced-learn Python package (version 0.6.2) [61]. The random forest classifier was implemented by the scikit-learn Python package (version 0.22.1) [62].

### Application on paroxysmal AF patients

To apply our trained classifier to predict patient’s AF mechanism (Fig 1 Step 4), data of 56 paroxysmal AF patients (two patients were considered borderline paroxysmal/persistent AF patients), including pre-operative BSPM signals from a 252-lead vest (CardioInsight, Medtronic, MN), as well as their clinical follow-up records, were exported from the Haut-Lévêque Cardiology Hospital (Bordeaux, France). All anti-arrhythmic medications were stopped 48 hours prior to the catheter ablation, and diltiazem was given to patients before the procedure to slow the atrioventricular conduction. During the procedure, AF was first induced with burst pacing, and PV isolation was performed. After the isolation, if the sinus rhythm or atrial tachycardia was not restored, sites of AF drivers (foci and/or stable rotor cores) mapped by the catheter and the ECGi solution by CardioInsight, were ablated. For all patients, AF was terminated at the end of all procedures.

The BSPM signals were acquired at a sampling rate of 1 kHz. QRS complex was extracted by the Pan-Tompkins algorithm [63]. T wave was extracted automatically by identifying the segment with a large variance over the signals of all channels on the segment between two QRS complexes. Only f-wave segments longer than 800 ms were included for analysis. We excluded three patients under 30 years old, as well as one patient who had only one f-wave segment recorded. The baseline population characteristics of the cohort included in the study are summarized in S5 Table. 13±4 segments were analyzed for each patient. All signals were first filtered by a standard 2–30 Hz second-order Butterworth filter to remove high-frequency noise and baseline wandering, and then were normalized channel-wise. We computed the signal-level mechanism for each signal as in Table 4. The patient-level mechanisms were obtained by aggregating all signal-level mechanisms of each patient.

**Table 4:** Computation of signal-level mechanisms from the positive (“+”) or negative (“−”) prediction outputs of our classifiers. Note that FS location classifier outputs whether the FS is on RA.

Mechanism	Classifier outputs		
	FS presence	AF sustainability	FS location
Initiator-type FS from LA/PVs	+	+	−
Initiator-type FS from RA	+	+	+
Driver-type FS from LA/PVs	+	−	−
Driver-type FS from RA	+	−	+
Sustained AF without FS	−	+	+/−
Neither AF nor FS	−	−	+/−

## Conclusion

To identify AF ablation targets from body surface potentials, we generated a large variety of AF episodes driven by FS and reentrant sources. We then determined the AF sustainability after removing FS. The FS and arrhythmogenic substrate were identified by a random forest classifier, with features of CL, periodicity as well as the signal contribution from the first source, extracted from body surface potentials using SO-BSS. From the computer models, we showed that longer rotor duration during FS episodes, which indicated an arrhythmogenic substrate for AF, was correlated with higher AF sustainability after FS removal with an AUROC of 0.83. With this biophysical basis, our classifiers were able to accurately predict the FS existence and location, and AF sustainability with robustness to both ECGs and BSPMs and inter-patient atrial variability. The mechanism prediction of FS presence and AF sustainability was also robust to extreme variations in vest placements. On 52 paroxysmal AF patients going through procedures based mainly on PV isolation, our classifiers predicted a patient subgroup achieving improved two-to-three-year AF-free rates (p-value < 0.01, logrank tests), by estimating patient AF mechanisms using their pre-operative BSPMs. Our study demonstrates the potential for advancing the mechanism-targeted treatment in AF patients, as well as detecting malignant ectopic beats with likely AF progression in the general population.

## Supporting information

S1 Appendix Implementation of SO-BSS for atrial source extraction. (PDF)

S1 Fig Effects of removing the FS for (a) the initiator-type FS with a CL of 180 ms in Fig 3a, where the AF continued after pacing stopped, and for (b) the driver-type FS with a CL of 210 ms in Fig 3b, where AF died out from 2400 ms, on the atrial mesh of Patient 2. The red stars mark the locations of the FS activated right before 2000 ms (at 1980 ms for (a) and at 1890 ms for (b)). The red arrows showed the movement of rotor wavefronts. The time was counted from the first pacing of the FS. A macro-reentry going through the coronary sinus can be seen from 2300–2700 ms on (a).
(TIF)

S2 Fig Simulations of reentrant AFs, including (a) rotor and (b) reentry with a conduction block of radius 1 cm on the atrial mesh of Patient 2. The red arrows indicate the directions of wavefront propagation. The time was counted from the initiation of the AF. The rotational sources can be clearly seen.
(TIF)

S3 Fig Simulation of the effect of ACh, on the atrial mesh of Patient 2. The red arrows indicated the directions of wavefront propagation. The red arrows indicate the directions of wavefront propagation. The time was counted from the initiation of AF. A local rotor appeared close to the base of the posterior left atrial wall where ACh islands were distributed. The potentials were also more heterogeneous with the introduction of ACh.
(TIF)

S4 Fig V1-lead ECG and SO-BSS sources extracted from the BSPM with *K* = 10 of an initiator-type FS (CL = 180 ms) at an LA focal site (*α*_*LA*_ = 0.2, *β*_*LA*_ = 0.2) on the atrial mesh of Patient 2, the same as Fig 3a, together with the ACF and the FFT power spectral density. The first 10 rows show the top 10 sources *s*_*i*_ as ranked by their eigenvalues, respectively, with the bottom row showing the V1-lead ECG. The first column shows the signal amplitude over time. The second column shows the value of ACF(*s*_*i*_) over time-lags up to 500 ms, ACF_*i*_, where red bars mark the CL_*i*_ with labels of CL_*i*_ and MaxAC_*i*_ outside and inside the bracket, or CL_*V*1_ and MaxAC_*V*1_, and shading shows the 95% confidence interval of the ACF. The third column shows the FFT power spectral density PSD_*i*_ or PSD_*V*1_, with the DF indicated by a red bar. a.u.: arbitrary unit.
(TIF)

S5 Fig V1-lead ECG and SO-BSS sources extracted from the BSPM with *K* = 10 of a driver-type FS (CL = 210 ms) at an LA focal site (*α*_*LA*_ = 0.2, *β*_*LA*_ = 0.2) on the atrial mesh of Patient 2, the same as the case of Fig 3b, together with the ACF and the FFT power spectral density. The legend is the same as S4 Fig.
(TIF)

S6 Fig V1-lead ECG and SO-BSS sources extracted from the BSPM with *K* = 10 of a reentrant AF initiated around the LA site without a non-excitable core at (*α*_*LA*_ = 0.2, *β*_*LA*_ = 0.2) on the atrial mesh of Patient 2, the same as the case of S2(a) Fig, together with the ACF and the FFT power spectral density. The legend is the same as S4 Fig.
(TIF)

S7 Fig Simulated ECG signals of (a) an initiator-type FS, (b) a driver-type FS, and (c) a reentrant AF, corresponding to Fig 3a and 3b, and S2(a) Fig, respectively, after channel-wise normalization. a.u.: arbitrary unit.
(TIF)

S8 Fig A beeswarm plot of the estimated dominant CL and the ground-truth focal CL over driver-type FS and initiator-type FS, using *K* = 10 for SO-BSS from BSPM signals. Data points were collapsed where possible for better visualization.
(TIF)

S9 Fig An example ACh distribution with (a) two-dimensional mapping, and (b) three-dimensional representation on the atrial mesh of Patient 2. The other areas without ACh islands are marked in gray. As mentioned in the text, fixed numbers of 60, 80, and 100 2mm-radius ACh islands (in yellow) were randomly distributed at areas of *α*_*LA*_ of 0–0.33, 0.33–0.67 and 0.67–1 and *β*_*LA*_ of 0–1, respectively. Note that in (a), only the positions but not the sizes of ACh islands were indicated.
(TIF)

S10 Fig An example setup to initiate a reentrant source around an LA site (*α*_*LA*_ = 0.2, *β*_*LA*_ = 0.2) on the atrial mesh of Patient 2. The other areas without the moderation of the initial states are marked in gray. To initiate a reentry on the LA, the LA body was split into 48 sectors, marked by 48 different colors in (b), around a non-excitable core. In this case, the non-excitable core has a radius of 0. The initial states were generated from an action potential after a complete depolarization of a cell, with selected states shown in (a). We provided example code to generate these initial states in https://doi.org/10.5281/zenodo.5105725.
(TIF)

S1 Video Video of simulations on the atrial mesh of Patient 2 of an initiator-type FS with a CL of 180 ms at an LA focal site (the same as Fig 3a). The white spheres below the right PVs mark the cores of sustained rotors.
(MP4)

S2 Video Video of simulations on the atrial mesh of Patient 2 of a driver-type FS with a CL of 210 ms at an LA focal site (the same as Fig 3b). There was no sustained rotor induced.
(MP4)

S1 Table The increase in AF inducibility from FS by adding ACh regulation, tested by one-sided proportion z-tests and grouped by focal CL and location. +ACh and −ACh denote with and without ACh, respectively. The blank entries represent either p-value > 0.05, or not applicable due to empty sample size.
(PDF)

S2 Table Means and standard deviations (s.d.) of AF inducibility (mean±s.d.%), as well as testing accuracy (mean±s.d.%) of leave-one-patient-out cross validation on the AF sustainability classification evaluated on all FS-driven episodes, using features of SO-BSS, AFFT_2*DF*_ and NDI, grouped by focal CL (ms). Bold fonts mark the highest scores and the corresponding features. The parentheses mark signals used. SO-BSS took *K* = 10 sources as an input parameter.
(PDF)

S3 Table Evaluation of dominant CL estimation and classifiers, with 252-lead vest variations of 5 and 10 cm in translation and 10 and 20 degree in rotation, on the dataset of Patient 2, using *K* = 10 and BSPM. Absolute dominant CL difference refers to the absolute difference in the dominant CLs between the original setting and the variation, for which the mean and the standard deviation (s.d.) are shown.
(PDF)

S4 Table Statistics of patient atrial meshes for simulations. No.: Number of. s.d: standard deviation. Min: minimum. Max: maximum.
(PDF)

S5 Table Baseline population characteristics of the included paroxysmal AF patients. (PDF)
