# Supplementary material for: Detection of focal source and arrhythmogenic substrate from body surface potentials to guide atrial fibrillation ablation
Source: PLoS Comput Biol. 2022 Mar 21;18(3):e1009893. doi: 10.1371/journal.pcbi.1009893 (PMC8970486; doi:10.1371/journal.pcbi.1009893)
Supplement: S1 Appendix — (PDF) [file pcbi.1009893.s001.pdf]

Implementation of SO-BSS for atrial source extraction from body surface potentials

Here we present atrial source extraction using an SO-BSS technique based on the algorithm SOBI [1]. The N-channel T-length body surface potentials, $\mathbf{X} = [\mathbf{x}_1, \mathbf{x}_2, \ldots, \mathbf{x}_N]^T$ of shape $N \times T$, were assumed to have arisen from a linear mixture of K equivalent atrial sources $\mathbf{S} = [\mathbf{s}_1, \mathbf{s}_2, \ldots, \mathbf{s}_K]^T$ of shape $K \times T$, where a linear relationship was assumed between the sources and the signals.

The signals were first normalized channel-wise to allow a similar signal amplitude between different channels. Afterwards, K channels with K largest eigenvalues through singular value decomposition on $\mathbf{X}$ were preserved, to reduce the computational burden in the following steps. The signals were then whitened to be $\hat{\mathbf{X}} = [\hat{\mathbf{x}}_1, \hat{\mathbf{x}}_2, \ldots, \hat{\mathbf{x}}_K]^T$ to remove correlation between channels. The transformation between the sources and the whitened signals involves a matrix $\mathbf{W}$ with shape $K \times K$:

$$\mathbf{S} = \mathbf{W}^T \hat{\mathbf{X}} \tag{1}$$

To estimate the second-order statistics of the signals, a whitened autocovariance matrix $C_x(\tau)$ with time-lag τ was defined.

$$C_x(\tau) = \hat{\mathbf{X}}(t+\tau)\hat{\mathbf{X}}(t)^T$$

An iterative algorithm to perform joint diagonalization [1] was applied to calculate K joint orthonormal eigenvectors $\{\tilde{\mathbf{w}}\}$ for all whitened autocovariance matrices $\{C_x(\tau) | \tau \in T_{FS}\}$, which constitute the columns of $\mathbf{W}$.

The extracted source of SO-BSS may also be its own harmonics. To best estimate the true dominant CL, we therefore set $T_{FS} = \{140, 141, \ldots, 370\}$ ms as the search range of source CLs. The upper bound of T_{FS} was set to cover all possible 2:1 conduction block conditions from FS with CL up to 185 ms, as the effective CLs of these FS on the surface signals were double the value of the focal CL. The lower bound marked the presumed minimal focal CL where the activation of FS could trigger 1:1 atrial responses.

The i-th source $\tilde{\mathbf{s}}_i$ and its corresponding (maximal) eigenvalue $\tilde{\lambda}_i$ obtained from the joint diagonalization can be estimated as:

$$\begin{aligned} \tilde{\mathbf{s}}_i &= \tilde{\mathbf{w}}_i^T \hat{\mathbf{X}} \\ \tilde{\lambda}_i &= \max_{\tau \in T_{FS}} \tilde{\mathbf{w}}_i^T C_x(\tau) \tilde{\mathbf{w}}_i \end{aligned}$$

We ranked the sources from high to low according to the eigenvalue for each source, $\tilde{\lambda}_i$. This constitutes our final estimated sources, $\mathbf{s}_1, \mathbf{s}_2, \ldots, \mathbf{s}_K$.

References

1. Belouchrani A, Abed-Meraim K, Cardoso JF, Moulines E. A blind source separation technique using second-order statistics. IEEE Trans Signal Process. 1997;45(2):434–444.
