# Supplementary figures and images for: Detection of focal source and arrhythmogenic substrate from body surface potentials to guide atrial fibrillation ablation
Source: PLoS Comput Biol. 2022 Mar 21;18(3):e1009893. doi: 10.1371/journal.pcbi.1009893 (PMC8970486; doi:10.1371/journal.pcbi.1009893)

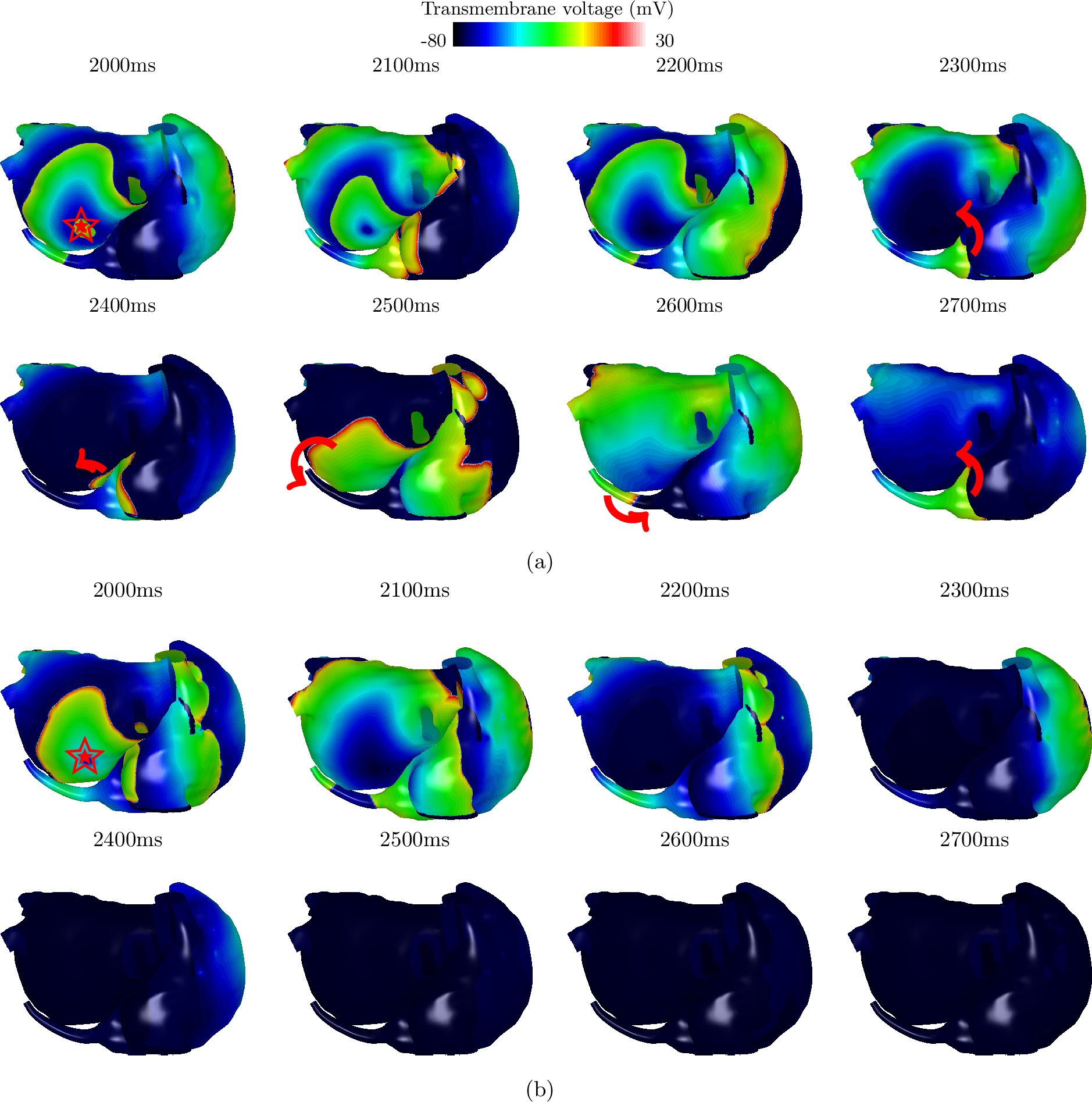

Supplement: S1 Fig — The red stars mark the locations of the FS activated right before 2000 ms (at 1980 ms for (a) and at 1890 ms for (b)). The red arrows showed the movement of rotor wavefronts. The time was counted from the first pacing of the FS. A macro-reentry going through the coronary sinus can be seen from 2300–2700 ms on (a). (TIF) [file pcbi.1009893.s002.tif]

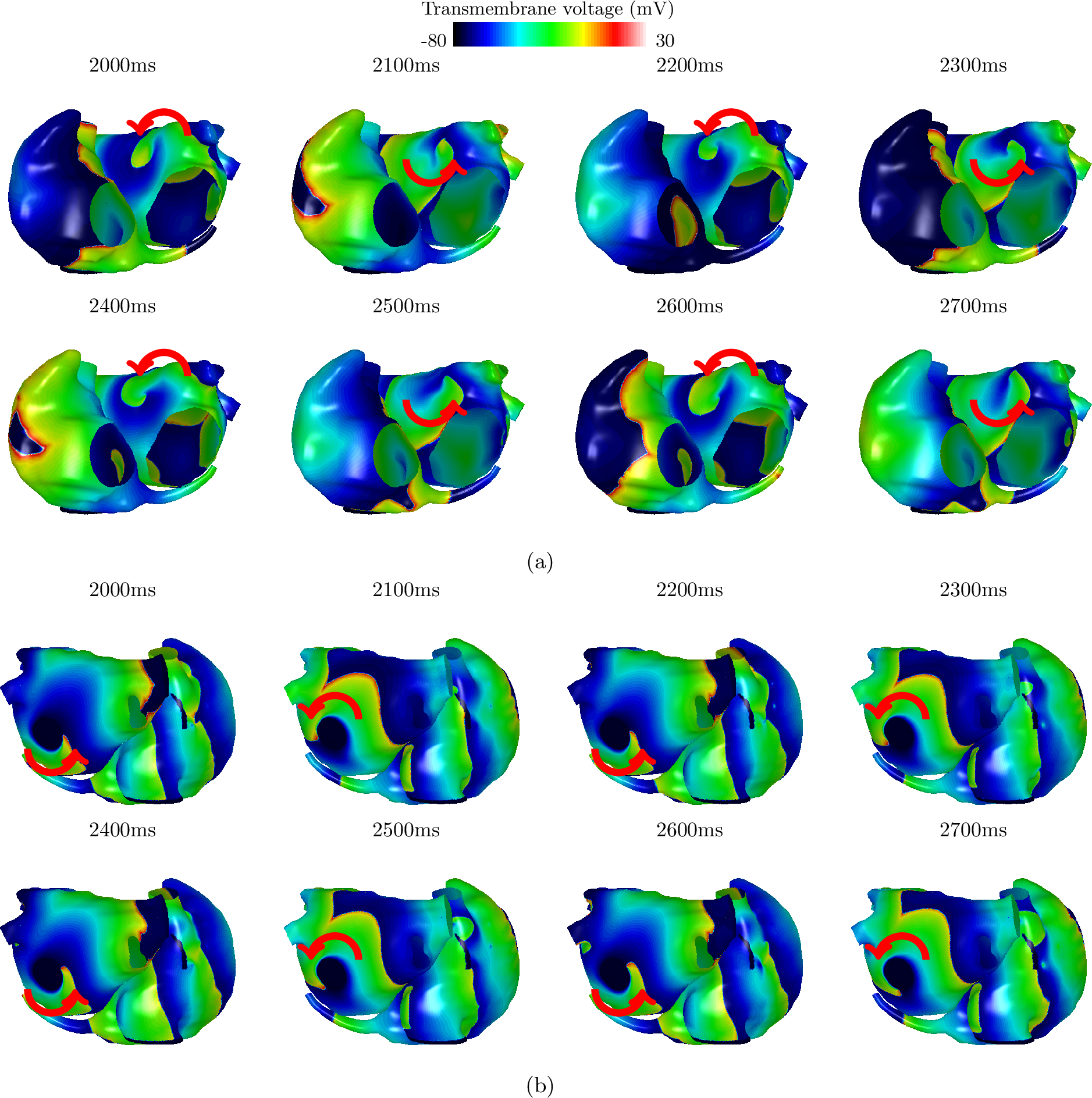

Supplement: S2 Fig — The red arrows indicate the directions of wavefront propagation. The time was counted from the initiation of the AF. The rotational sources can be clearly seen. (TIF) [file pcbi.1009893.s003.tif]

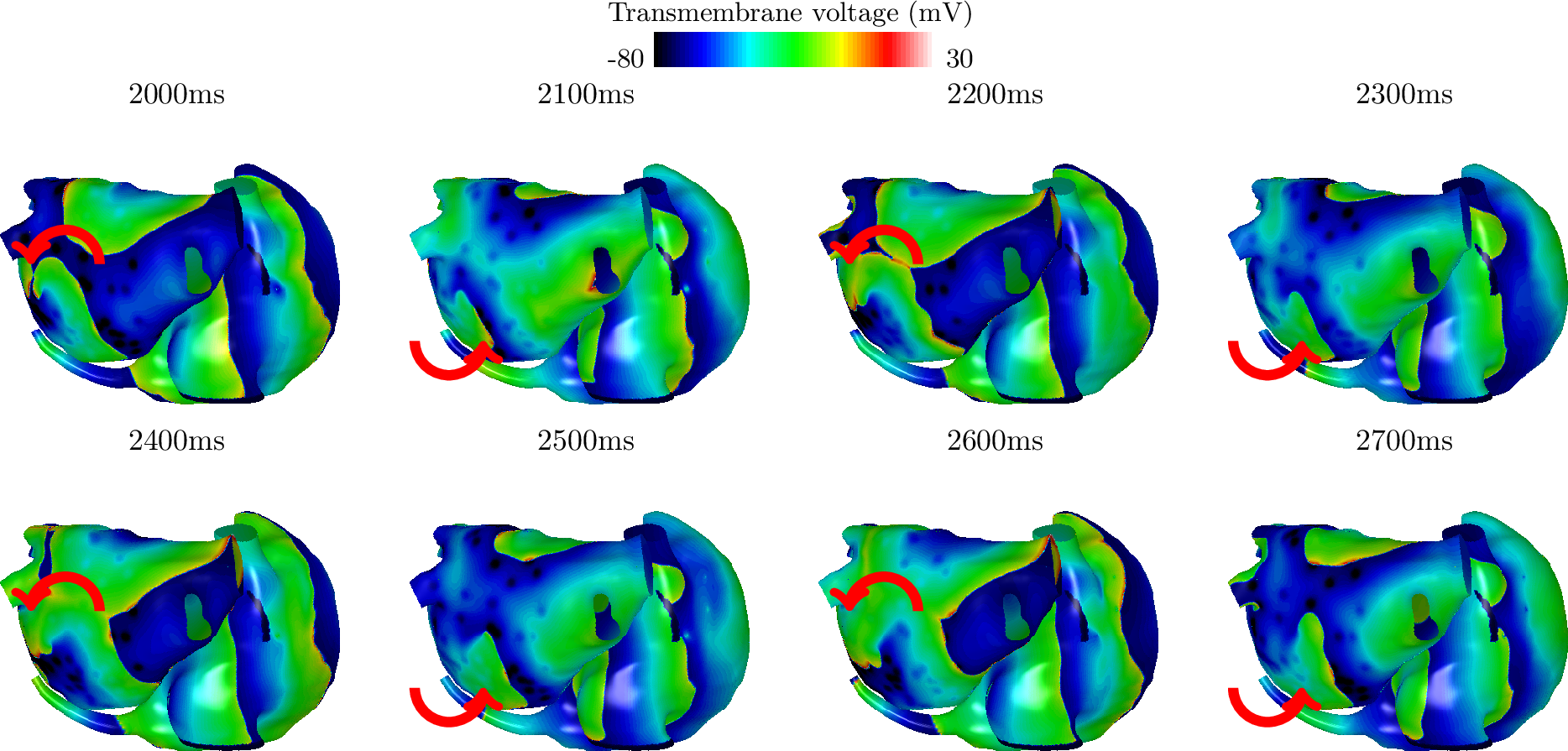

Supplement: S3 Fig — The red arrows indicated the directions of wavefront propagation. The red arrows indicate the directions of wavefront propagation. The time was counted from the initiation of AF. A local rotor appeared close to the base of the posterior left atrial wall where ACh islands were distributed. The potentials were also more heterogeneous with the introduction of ACh. (TIF) [file pcbi.1009893.s004.tif]

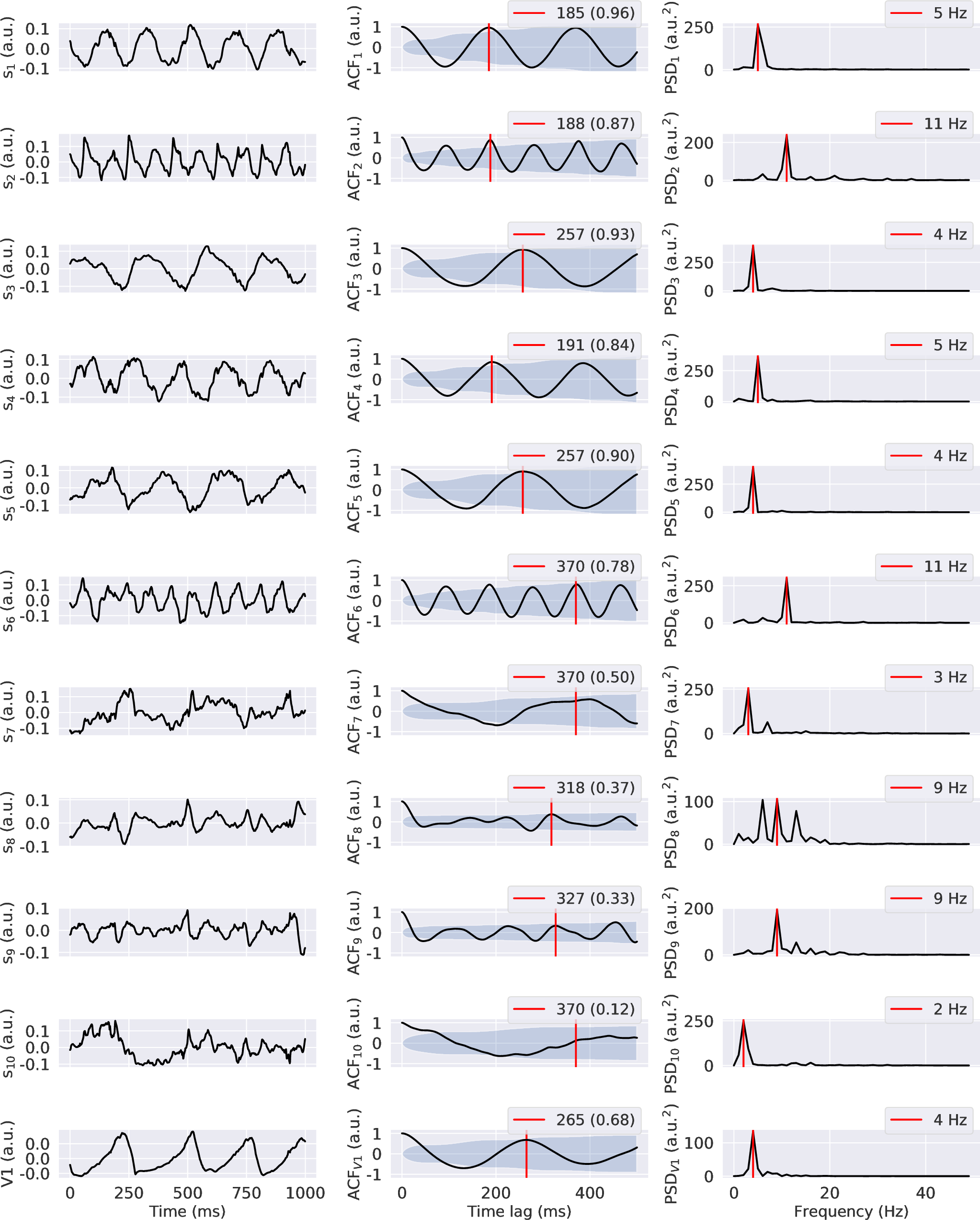

Supplement: S4 Fig — The first 10 rows show the top 10 sources si as ranked by their eigenvalues, respectively, with the bottom row showing the V1-lead ECG. The first column shows the signal amplitude over time. The second column shows the value of ACF(si) over time-lags up to 500 ms, ACFi, where red bars mark the CLi with labels of CLi and MaxACi outside and inside the bracket, or CLV1 and MaxACV1, and shading shows the 95% confidence interval of the ACF. The third column shows the FFT power spectral density PSDi or PSDV1, with the DF indicated by a red bar. a.u.: arbitrary unit. (TIF) [file pcbi.1009893.s005.tif]

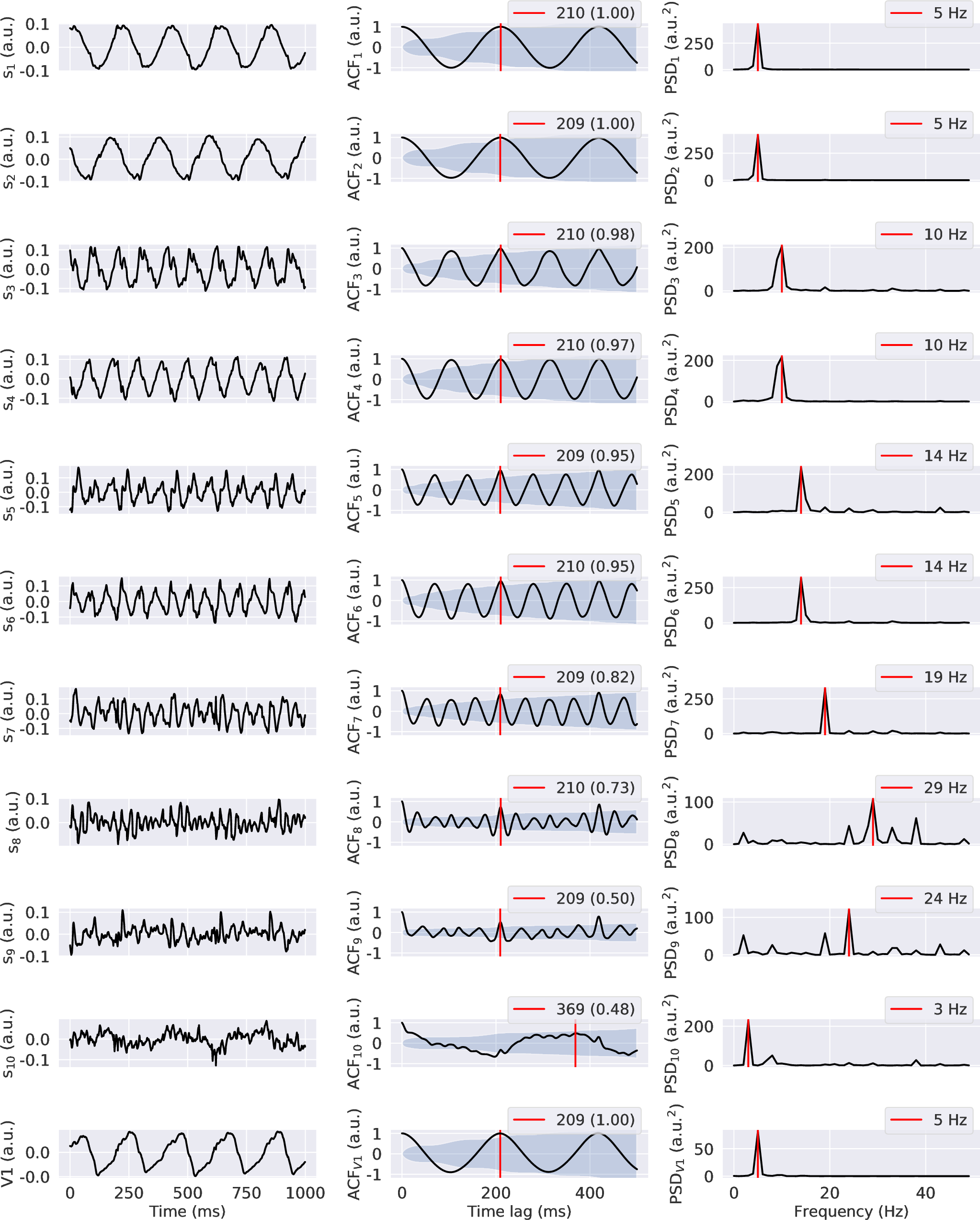

Supplement: S5 Fig — The legend is the same as S4 Fig. (TIF) [file pcbi.1009893.s006.tif]

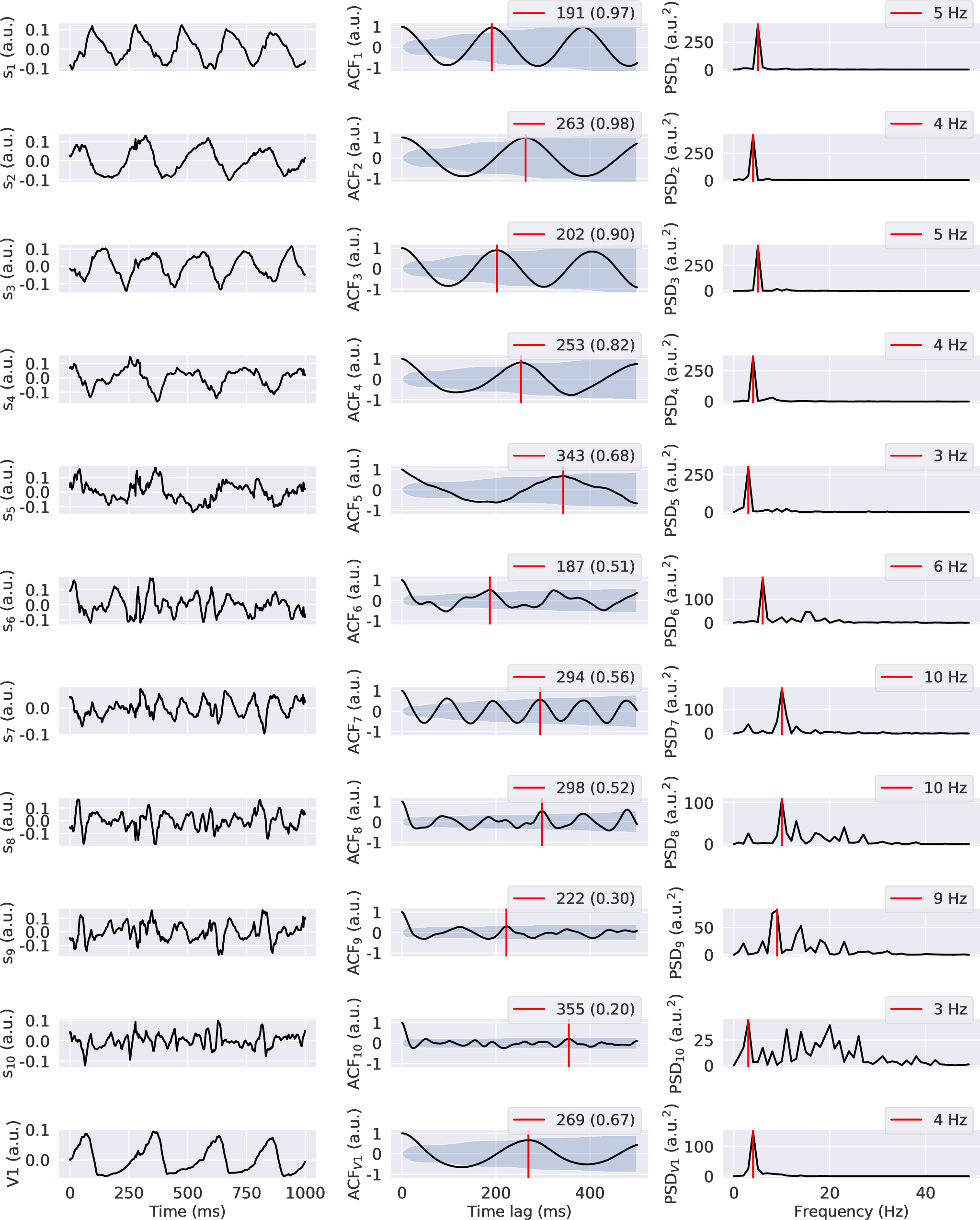

Supplement: S6 Fig — The legend is the same as S4 Fig. (TIF) [file pcbi.1009893.s007.tif]

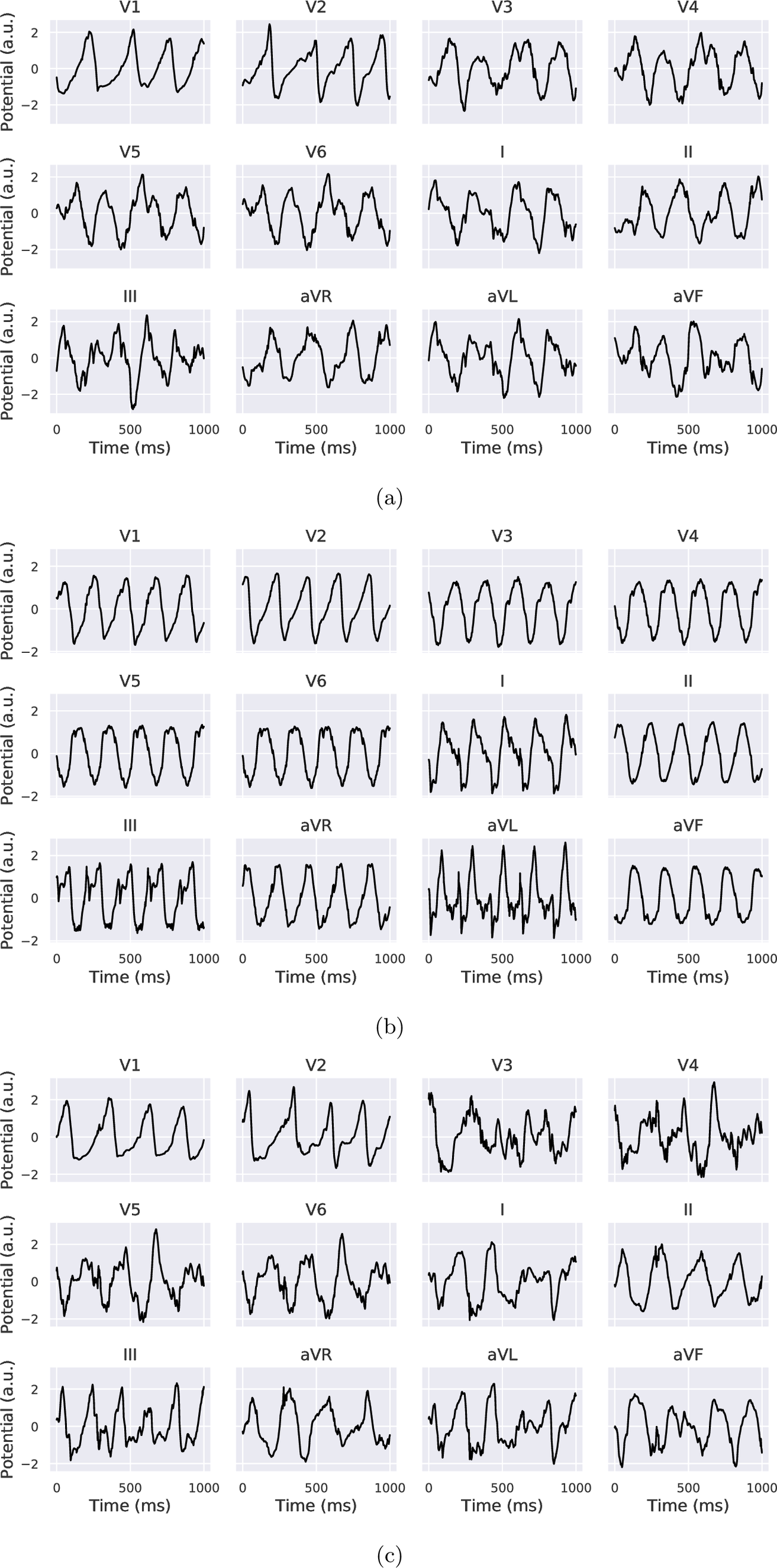

Supplement: S7 Fig — a.u.: arbitrary unit. (TIF) [file pcbi.1009893.s008.tif]

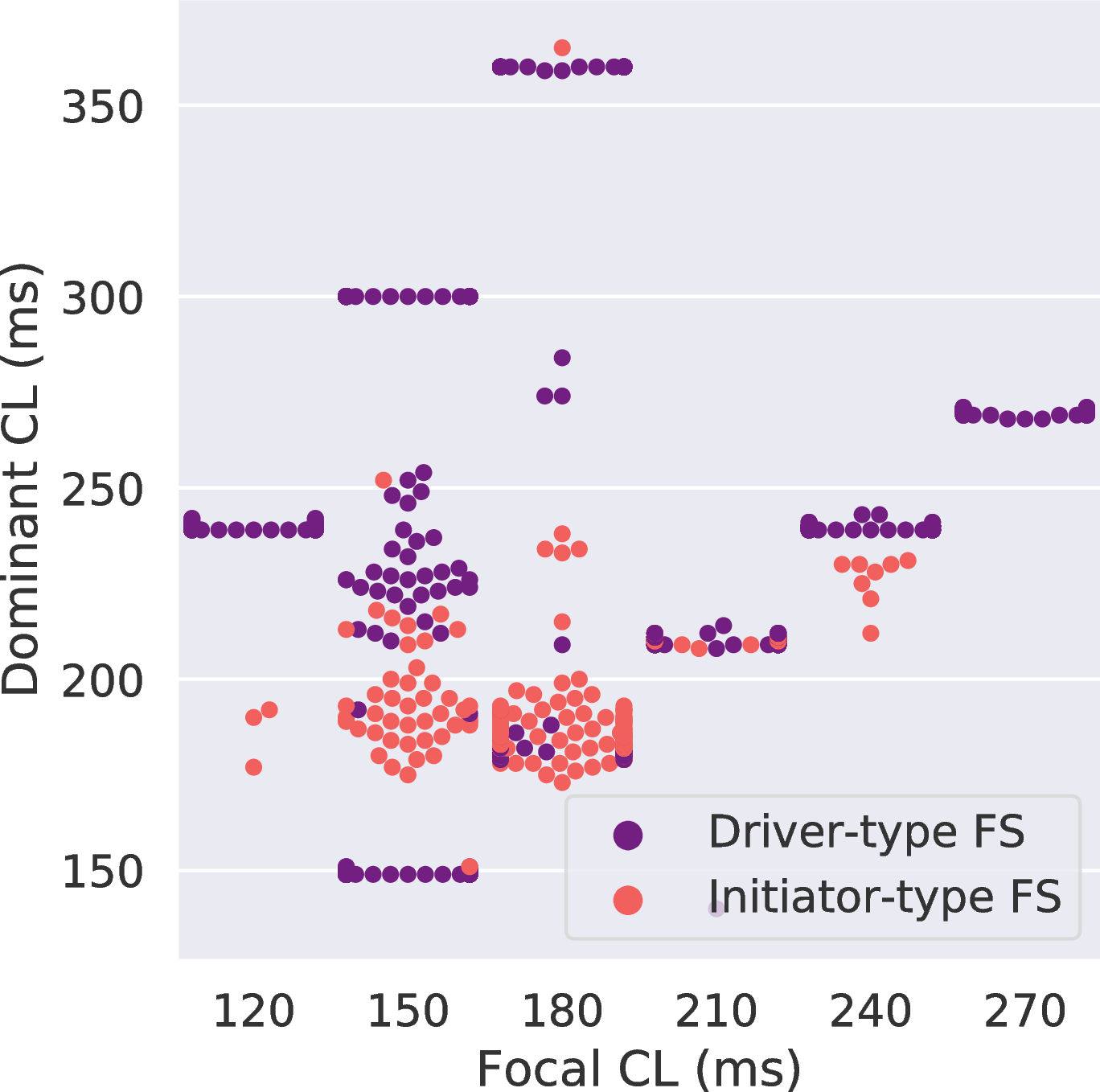

Supplement: S8 Fig — Data points were collapsed where possible for better visualization. (TIF) [file pcbi.1009893.s009.tif]

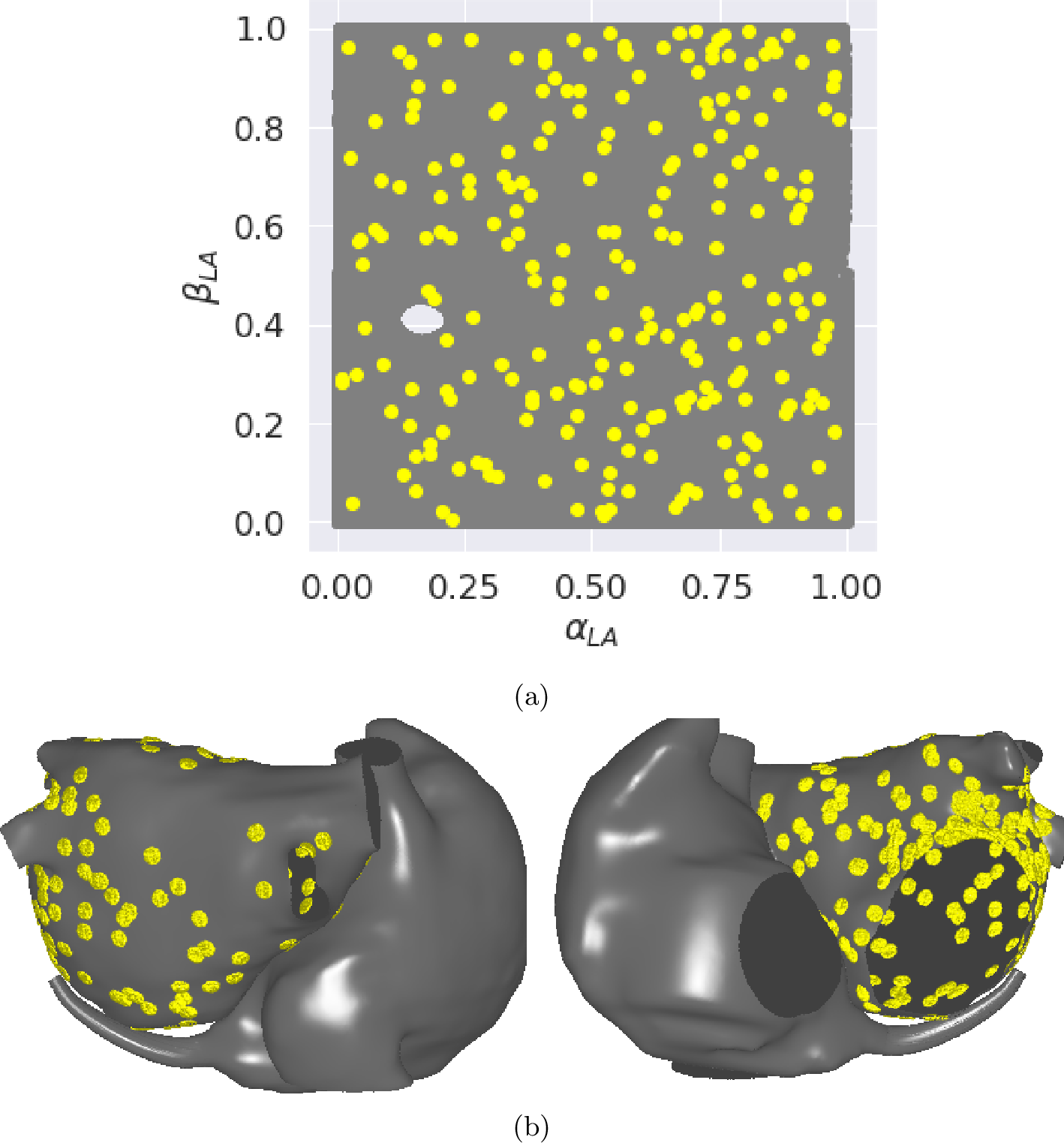

Supplement: S9 Fig — The other areas without ACh islands are marked in gray. As mentioned in the text, fixed numbers of 60, 80, and 100 2mm-radius ACh islands (in yellow) were randomly distributed at areas of αLA of 0–0.33, 0.33–0.67 and 0.67–1 and βLA of 0–1, respectively. Note that in (a), only the positions but not the sizes of ACh islands were indicated. (TIF) [file pcbi.1009893.s010.tif]

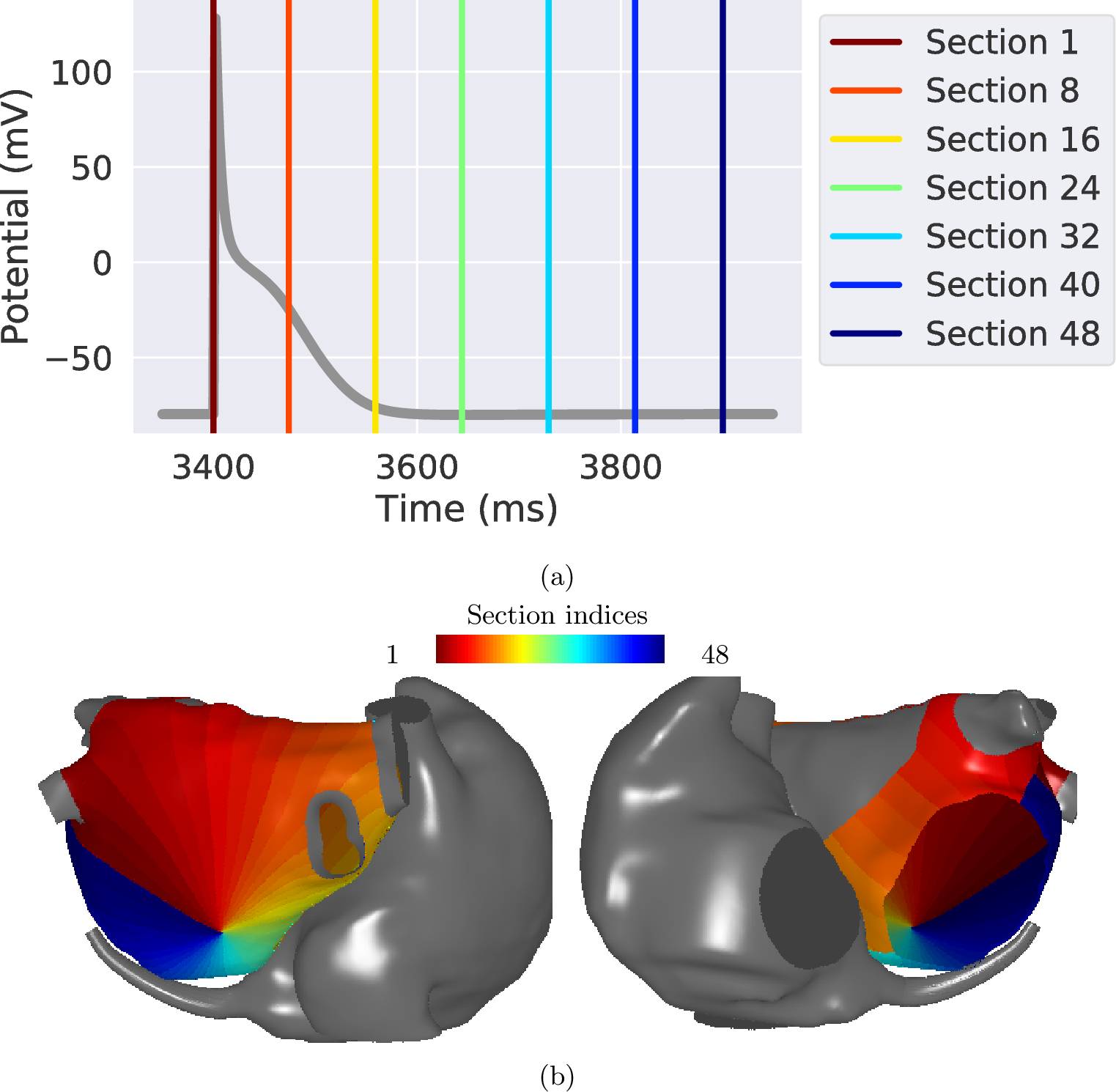

Supplement: S10 Fig — The other areas without the moderation of the initial states are marked in gray. To initiate a reentry on the LA, the LA body was split into 48 sectors, marked by 48 different colors in (b), around a non-excitable core. In this case, the non-excitable core has a radius of 0. The initial states were generated from an action potential after a complete depolarization of a cell, with selected states shown in (a). We provided example code to generate these initial states in https://doi.org/10.5281/zenodo.5105725. (TIF) [file pcbi.1009893.s011.tif]
