# Supplementary material for: Detection of focal source and arrhythmogenic substrate from body surface potentials to guide atrial fibrillation ablation
Source: PLoS Comput Biol. 2022 Mar 21;18(3):e1009893. doi: 10.1371/journal.pcbi.1009893 (PMC8970486; doi:10.1371/journal.pcbi.1009893)
Supplement: S1 Table — +ACh and −ACh denote with and without ACh, respectively. The blank entries represent either p-value > 0.05, or not applicable due to empty sample size. (PDF) [file pcbi.1009893.s014.pdf]

Focal CL (ms)	FS location	AF inducibility (+ACh, −ACh)	p-value (+ACh > −ACh)
120	PV	0.05, 0	
	LA	0.03, 0.01	
	RA	0, 0	
150	PV	0.70, 0.30	**$<$0.01**
	LA	0.40, 0	**$<$0.0001**
	RA	0, 0	
180	PV	0.95, 0.95	
	LA	0.95, 0.81	**$<$0.01**
	RA	0.01, 0.01	
210	PV	0.35, 0.25	
	LA	0.36, 0.17	**$<$0.01**
	RA	0.21, 0.26	
240	PV	0, 0	
	LA	0, 0	
	RA	0.05, 0.05	
270	PV	0, 0	
	LA	0, 0	
	RA	0, 0	
