# Supplementary material for: Detection of focal source and arrhythmogenic substrate from body surface potentials to guide atrial fibrillation ablation
Source: PLoS Comput Biol. 2022 Mar 21;18(3):e1009893. doi: 10.1371/journal.pcbi.1009893 (PMC8970486; doi:10.1371/journal.pcbi.1009893)
Supplement: S2 Table — Bold fonts mark the highest scores and the corresponding features. The parentheses mark signals used. SO-BSS took K = 10 sources as an input parameter. (PDF) [file pcbi.1009893.s015.pdf]

CL	AF inducibility	**SO-BSS (BSPM)**	$AFFT_{2DF}$ (V1 ECG)	NDI (BSPM)
120	1.9±0.8	**98.8±1.2**	60.9±11.1	67.3±8.9
150	14.3±2.1	**88.6±3.2**	71.7±3.6	62.9±8.6
180	49.1±2.2	**97.2±3.0**	63.5±3.9	69.4±5.2
210	25.7±18.5	**65.9±12.5**	49.2±3.3	53.4±16.6
240	3.8±0.9	**98.9±1.9**	61.7±5.4	65.6±7.8
270	0	**99.4±1.3**	54.8±4.9	60.5±3.8
