# Supplementary material for: Detection of focal source and arrhythmogenic substrate from body surface potentials to guide atrial fibrillation ablation
Source: PLoS Comput Biol. 2022 Mar 21;18(3):e1009893. doi: 10.1371/journal.pcbi.1009893 (PMC8970486; doi:10.1371/journal.pcbi.1009893)
Supplement: S3 Table — Absolute dominant CL difference refers to the absolute difference in the dominant CLs between the original setting and the variation, for which the mean and the standard deviation (s.d.) are shown. (PDF) [file pcbi.1009893.s016.pdf]

		Original	Rotate around z axis (degree)				Translate in x direction (cm)				Translate in y direction (cm)				Translate in z direction (cm)			
			-20	-10	10	20	-10	-5	5	10	-10	-5	5	10	-10	-5	5	10
Absolute dominant CL difference	Mean (ms)	0.0	3.05	2.41	3.39	4.35	2.99	2.53	3.76	3.14	4.65	2.27	4.78	7.09	2.92	2.06	1.21	2.91
	s.d. (ms)	0.0	20.15	17.35	20.57	24.18	17.49	17.26	22.21	20.15	22.78	15.44	23.83	30.02	18.94	16.14	11.49	18.48
FS presence	Accuracy (%)	91.3	90.8	91.6	90.8	90.7	90.2	91.6	91.5	90.8	90.5	90.7	90.7	91.9	91.8	91.0	91.6	90.8
	Precision (%)	97.7	97.6	97.9	97.4	97.1	97.1	97.7	97.7	98.7	93.1	96.4	96.6	94.5	97.7	97.4	97.7	96.7
	Recall (%)	89.3	88.6	89.5	88.8	88.8	88.1	89.8	89.5	87.6	92.9	89.5	89.3	93.6	90.0	89.0	89.8	89.5
AF sustainability	Accuracy (%)	93.7	92.8	93.4	92.6	92.9	92.9	92.9	93.4	93.2	93.4	92.4	93.1	92.1	92.8	92.9	93.6	92.8
	Precision (%)	94.0	92.6	93.6	91.6	92.3	92.9	92.6	92.7	92.6	95.0	92.8	93.6	93.4	91.6	92.6	92.7	92.9
	Recall (%)	91.6	90.9	91.2	91.6	91.6	90.9	91.2	92.3	92.0	89.8	89.8	90.5	88.3	92.0	91.2	92.7	90.5
AF sustainability (from FS)	Accuracy (%)	90.7	89.5	90.5	89.0	89.8	89.8	89.8	90.2	90.2	90.5	89.5	89.8	88.8	89.3	89.5	90.7	89.5
	Precision (%)	75.8	71.0	74.6	68.5	70.8	72.1	71.4	72.2	72.2	78.0	71.6	73.4	71.7	68.9	71.0	73.0	71.6
	Recall (%)	68.5	67.1	68.5	68.5	69.9	67.1	68.5	71.2	71.2	63.0	65.8	64.4	58.9	69.9	67.1	74.0	65.8
Focal site region (from FS)	Accuracy (%)	83.6	78.1	79.8	79.5	73.6	59.8	75.5	65.5	53.6	75.7	76.9	67.6	54.0	57.6	71.0	66.2	63.1
	Precision (%)	87.7	82.1	84.1	81.9	77.9	62.9	81.1	73.3	45.2	81.7	81.0	73.3	48.5	56.1	71.1	82.9	89.4
	Recall (%)	74.5	66.7	68.8	70.8	58.9	29.2	60.4	38.5	7.3	60.4	64.6	45.8	8.3	33.3	61.5	32.8	21.9
