# Supplementary material for: Detection of focal source and arrhythmogenic substrate from body surface potentials to guide atrial fibrillation ablation
Source: PLoS Comput Biol. 2022 Mar 21;18(3):e1009893. doi: 10.1371/journal.pcbi.1009893 (PMC8970486; doi:10.1371/journal.pcbi.1009893)
Supplement: S4 Table — No.: Number of. s.d: standard deviation. Min: minimum. Max: maximum. (PDF) [file pcbi.1009893.s017.pdf]

	No. nodes	Triangular elements					Linear elements				
		No. elements	Edge lengths (μm)				No. elements	Edge lengths (μm)			
			Mean	s.d.	Min	Max		Mean	s.d.	Min	Max
Mesh 1	492799	97421	352	74	58	1701	1191111	111	79	94	11465
Mesh 2	595997	1178840	351	73	57	1595	1445899	110	99	100	11016
Mesh 3	402343	793683	348	60	49	1069	963281	110	74	100	8584
Mesh 4	528589	1045271	349	66	58	1701	1270575	108	72	94	11465
Mesh 5	427664	844727	352	58	9	911	1040902	109	88	64	11286
