# Supplementary material for: Detection of focal source and arrhythmogenic substrate from body surface potentials to guide atrial fibrillation ablation
Source: PLoS Comput Biol. 2022 Mar 21;18(3):e1009893. doi: 10.1371/journal.pcbi.1009893 (PMC8970486; doi:10.1371/journal.pcbi.1009893)
Supplement: S5 Table — (PDF) [file pcbi.1009893.s018.pdf]

	n=52 (%), or mean±s.d.
Gender	
Female	9 (17.3)
Male	43 (82.7)
Age (years)	58.3±11.6
Body mass index	26.8±4.1
AF total duration (years)	7.1±6.2
Maximum AF duration (hours)	36±30
$CHADS_2$ score (sample=37)	
0	24 (64.9)
1	10 (27.0)
2	2 (5.4)
5	1 (2.7)
Echocardiographic parameters	
Left ventricle ejection fraction (%)	59±8
Left atrial surface (cm^2)	23±9
Usage of $\geq$ 1 anti-arrhythmic drugs (sample=36)	30 (83.3)
Patients with $\geq$ 1 DC cardioversion (sample=37)	3 (8.1)
Patients with amiodarone (sample=37)	6 (16.2)
Procedure time (hours)	3.9±1.7
Other diseases	
Hypertension	15 (28.8)
Diabetes	2 (3.8)
Stroke	3 (5.8)
